# Actin Depolymerizing Factor Modulates Rhizobial Infection and Nodule Organogenesis in Common Bean

**DOI:** 10.3390/ijms21061970

**Published:** 2020-03-13

**Authors:** Yolanda Ortega-Ortega, Janet Carrasco-Castilla, Marco A. Juárez-Verdayes, Roberto Toscano-Morales, Citlali Fonseca-García, Noreide Nava, Luis Cárdenas, Carmen Quinto

**Affiliations:** 1 Departamento de Biociencias y Agrobiotecnología, Centro de Investigación en Química Aplicada-CONACYT, Saltillo 25294, Coahuila, Mexico; yolanda.ortega@ciqa.edu.mx; 2 Instituto Politécnico Nacional, Centro de Estudios Científicos y Tecnológicos 17 León, León 37358, Guanajuato, Mexico; teyane85@gmail.com; 3 Departamento de Docencia, Universidad Autónoma Agraria Antonio Narro, Saltillo 25315, Coahuila, Mexico; relf125@hotmail.com; 4 Department of Plant Biology, College of Biological Sciences, University of California, Davis, CA 95616, USA; rtoscano@umd.edu; 5 Departamento de Biología Molecular de Plantas, Instituto de Biotecnología, UNAM, Cuernavaca 62210, Morelos, Mexico; fonsecac@ibt.unam.mx (C.F.-G.); noreide@ibt.unam.mx (N.N.); luisc@ibt.unam.mx (L.C.)

**Keywords:** actin cytoskeleton, ADF, *Phaseolus vulgaris*, rhizobia, symbiosis, signaling

## Abstract

Actin plays a critical role in the rhizobium–legume symbiosis. Cytoskeletal rearrangements and changes in actin occur in response to Nod factors secreted by rhizobia during symbiotic interactions with legumes. These cytoskeletal rearrangements are mediated by diverse actin-binding proteins, such as actin depolymerization factors (ADFs). We examined the function of an ADF in the *Phaseolus vulgaris*–rhizobia symbiotic interaction (*PvADFE*). *PvADFE* was preferentially expressed in rhizobia-inoculated roots and nodules. *PvADFE* promoter activity was associated with root hairs harbouring growing infection threads, cortical cell divisions beneath root hairs, and vascular bundles in mature nodules. Silencing of *PvADFE* using RNA interference increased the number of infection threads in the transgenic roots, resulting in increased nodule number, nitrogen fixation activity, and average nodule diameter. Conversely, overexpression of *PvADFE* reduced the nodule number, nitrogen fixation activity, average nodule diameter, as well as *NODULE INCEPTION* (*NIN*) and *EARLY NODULIN2* (*ENOD2*) transcript accumulation. Hence, changes in *ADFE* transcript levels affect rhizobial infection and nodulation, suggesting that ADFE is fine-tuning these processes.

## 1. Introduction

Legumes have the ability to establish a symbiotic association with Gram-negative soil bacteria belonging to several genera, including *Rhizobium*, *Bradyrhizobium*, *Sinorhizobium*, and *Azorhizobium*, commonly called rhizobia [1]. This mutualistic interaction is initiated by a molecular dialogue in which plant roots exude flavonoids that activate the expression of bacterial genes (*nod* genes) encoding proteins involved in the synthesis and secretion of lipochitooligosaccharides, called Nod factors (NFs). These signal molecules are specifically recognized by the root hair, where they activate a signaling pathway. The NFs perceived by legume root hairs trigger a variety of physiological responses, such as calcium fluxes, including perinuclear calcium oscillations, reactive oxygen species production, ion influxes and effluxes, cell-membrane depolarization, cytoplasm alkalinization; cytoskeletal rearrangements, and early nodulin gene expression changes [2,3,4,5,6,7]. These changes allow the bacteria to enter the root hair through a structure called an infection thread (IT) and simultaneously induce cortical cell divisions. When the ITs reach the nodule primordia cells, rhizobia are released into the host cell within a peribacteroid membrane; subsequently, they differentiate into nitrogen-fixing bacteroids [8,9]. After root hair curling, the rhizobia trapped in an infection chamber inside the root hair curl, which is followed by an invagination of the host cell plasma membrane and the subsequent formation of an IT [10]. The growing IT induces changes in the plasmodesmata, cytoskeleton and cell membrane and wall synthesis machineries [11,12,13]. 

Actin cytoskeleton rearrangements take place at different stages of the symbiotic interaction between rhizobia and their host plants, i.e., during root hair growth and curling, IT formation, bacterial internalization from root hairs to nodule cells, symbiosome organization within the infected cells of root nodules, and cell trafficking [3,14,15,16]. In response to NFs, the actin cytoskeleton changes within 5–10 min; the longitudinal bundles of actin filaments become fragmented and short and fine actin bundles accumulate in the apical and subapical region, and there is a rapid increase in the number of F-actin plus ends at the tips of the responding root hairs [3,17,18].

In nodule cells, the actin cytoskeleton encloses IT and infection droplets, guiding the elongation of IT and the rhizobial release. During later steps of cell colonization, actin microfilaments embraces the developing symbiosomes and, in a mature nodule, actin reorganizes in several short or dot-like actin filaments aligned with mature symbiosomes [19]. Actin cytoskeleton architecture and dynamics are temporally and spatially modulated by diverse actin-binding proteins (ABPs), including profilin, villin, formin, ARPs, and actin depolymerizing factor (ADF/cofilin) [20]. The functions of ABPs include G-actin sequestration, actin nucleation, polymerization, depolymerization, and stabilization of F-actin filaments [20,21]. In general, it is well known that in plant cells upon several stimuli, the actin remodeling and assembly is triggered by small GTPases (ROPs in plants) [22]. These ROPs can activate the nucleation promoting factors (NPFs) such as Wave Regulatory Complex (WRC) consisting of SCAR/WAVE, nap1, pir1, abi and brick, which are a WASP family members that induce actin nucleation and rearrangements via recruitment and activation of the Arp2/3 complex [15]. Then, the Arp2/3 complex (consisting of 7 subunits), induces F-actin formation and the turnover is promoted by ADF/cofilins [23]. In *Lotus japonicus*, mutants of two components of the SCAR/WAVE complex, *nap1* (for Nck-associated protein 1) and *pir1* (for 121F-specific p53 inducible RNA), result in disruption of actin rearrangements, short root hairs, impaired formation and progression of ITs into the root cortex, and uncolonized nodule primordia [15]. In fact, silencing of the gene *ARP3* causes defects in symbiosome development in *Medicago truncatula* nodules [24] and *L. japonicus* mutants in *SCARN* (encoding suppressor of cAMP receptor defect-nodulation, a protein that binds to the ARP3 complex), present a strong phenotype with reduced root hair growth and aberrant formation and progression of ITs, resulting in uninfected nodules [25]. These results add weight to the idea that actin assembly is an important player during the symbiotic interaction.

ADFs are a family of small proteins of approximately 15–22 kDa found in all eukaryotes [26]. They share a conserved structural motif known as the actin-depolymerizing factor homology (ADF-H) domain consisting of five β-strands surrounded by three or more α-helices [27,28]. These proteins regulate actin filament depolymerization through binding both G-actin and F-actin in a twisted region of the actin filament, which further increases the twisting of the neighboring region [29]. This over-twisting results in the severing of actin filaments into shorter fragments and enhanced dissociation of G-actin from the minus ends of actin filaments [28]. The binding between actin and ADFs is regulated by phosphorylation, pH, phosphoinositides, and Ca^2+^ signaling [30,31,32,33], demonstrating that ADF activity is highly regulated [20]. Whereas metazoan animals harbour 1–4 *ADF/cofilin* genes [34], plant *ADF* genes form a large family in angiosperms; for example, the *Arabidopsis thaliana* genome contains at least 11 members (AtADF1 to AtADF11) showing organ-specific expression patterns [35]. Although a role for ADFs in defense signaling following pathogen infection has been reported [36,37,38,39,40], information about legume ADFs and their relationship to the symbiotic process are lacking.

We previously demonstrated that actin dynamics and polymerization regulate the early infection process in *Phaseolus vulgaris* root hairs incubated with purified specific NFs [3,18]. These actin dynamics could be mediated by ADFs, which are involved in F-actin depolymerization. Herein, we examined the function of a *P. vulgaris* ADF during symbiotic interactions with rhizobia. We found that promoter activity *PvADFE* was detected in the rhizobially infected root hairs and vascular bundles of mature nodules. The participation of *PvADFE* in the common bean symbiosis with *Rhizobium tropici* by down-regulating or overexpressing *PvADFE* in transgenic composite plants revealed a role of ADFE likely fine-tuning nodulation.

## 2. Results

### 2.1. ADF Genes Constitute a Family of Nine Members in P. vulgaris

To investigate the participation of *P. vulgaris* ADF during symbiosis with *R. tropici*, first we searched the Phytozome v12 database (http://phytozome.jgi.doe.gov/pz/portal.html#) [41]. Nine ADF genes were identified in the *P. vulgaris* genome. The deduced protein sequences were arbitrarily denominated PvADFA through PvADFI. The size of the *PvADF* gene family in *P. vulgaris* is similar to that of other legumes (Table S1). Based on gene structure analysis, the coding region of each *PvADF* gene is organized in three exons (Figure S1A), similar to the two- to three-exon organization of *Glycine max* (Figure S1B), *A. thaliana*, and *Solanum lycopersicum ADF* genes [42,43]. In eight of the nine *PvADF* genes, the first or first few amino acids are encoded by a separate exon, as described for *A. thaliana ADF* genes [28]. The nucleotide identity among *PvADF* genes varied from 48.9% to 85.2% (Table S2), whereas the identity between deduced amino acid sequences of PvADF ranged from 40.0% to 89.5% (Table S3). Multiple sequence alignment of the deduced protein sequences of PvADF and Arabidopsis AthADF1 revealed several conserved residues (Figure S2A). Ser-6, identified in other ADFs as a phosphorylation site [44], is followed by a G-actin binding motif (amino acids 6, 7, 125, and 128). This actin-binding motif is accompanied by a second signature specific for F-actin binding, with the amino acids 82, 84, 98, 136, and 137 conserved [45]. In addition, PvADF proteins contain a predicted nuclear localization signal (amino acids 22–28) [46]. The short sequence Trp90 through Met102 is also highly conserved in all PvADF proteins and has been identified as a binding site for both actin and phosphatidylinositol 4,5-bisphosphate (PIP_2_) [47]. Next, we predicted the three-dimensional structure of PvADFE using the crystal structure of AthADF1, which shares 82% amino acid sequence identity (Table S3), as the template. The predicted tertiary structure of PvADFE is similar to that of Arabidopsis AthADF1 [48], showing three α-helices surrounded by five β-sheets and a putative actin-binding surface (Figure S2B), a feature conserved among ADFs that is critical for binding and/or depolymerization of actin filaments [48]. In addition, there is 82% amino acid sequence identity among AthADF1 and PvADFE, as noted in Table S3.

To study the relationship between PvADF proteins, full-length ADF sequences from legumes (*G. max*, *L. japonicus*, *M. truncatula*, and *Vigna unguiculata*) and nonlegumes (*A. thaliana*, *Oriza sativa*, and *Zea mays*) were used to reconstruct a maximum likelihood phylogenetic tree. In relation to the Arabidopsis *ADF* gene family, which is grouped phylogenetically into four ancient subclasses based on differential expression [35], PvADFD and PvADFE protein sequences and other legume ADFs clustered in a subclade (Figure S3) closely related to AthADF1 through AthADF4 (Arabidopsis subclass I), which showed high transcript levels in roots, seedlings, mature leaves, and flowers [35].

### 2.2. PvADF Genes Are Expressed in Roots and after Rhizobial Inoculation

In *A. thaliana*, transcript accumulation of certain *ADF* genes is especially high in particular tissues; for example, *AthADF8* and *AthADF11* transcripts are elevated in roots compared with other tissues [35,49]. We analyzed transcript abundance of the nine *PvADF* genes using RT-qPCR in root hairs, apices, and stripped roots from common bean. As shown in Figure S4, accumulation of *PvADF* transcripts showed different patterns in the tissues examined. Transcripts of *PvADFA* and *F* were barely detected in root sections, and *PvADFI* expression was not detectable. The remaining *PvADF* genes were expressed in all root sections, with considerably higher expression of *PvADFD* and *H* than the other genes. In root hairs, transcripts of *PvADFD*, *E*, and *H* were the most abundant. However, *PvADFD* transcript abundance was highest in all tissues tested.

To examine the expression patterns of individual *ADF* genes after rhizobial inoculation, we performed an in silico analysis based on RNA-sequencing data reported in the *Phaseolus vulgaris* Gene Expression Atlas (PvGEA, http://plantgrn.noble.org/PvGEA/). According to PvGEA, the nine *PvADF* genes are expressed in aerial tissues, seeds, roots, and nodules. Interestingly, the *PvADFE* gene has the highest transcript levels expression among *PvADF* genes in both inoculated roots and nodules, at 5 and 21 days post-inoculation (dpi); *PvADFA*, *B*, *C*, and *F* are barely detected in all tissues tested, whereas *PvADFG*, *H*, and *I* are expressed weakly (Figure S5). Besides, *ADFE* encodes a protein of interest previously identified by our group using a phosphoproteomic approach in which the relative abundance of *PvADFE* increased after NF treatment in bean roots at 30 min. Then *PvADFE* was selected for further analysis during symbiosis with *R. tropici*.

### 2.3. PvADFE Promoter Activity in Transgenic P. vulgaris after Rhizobial Inoculation

**Figure 1:**
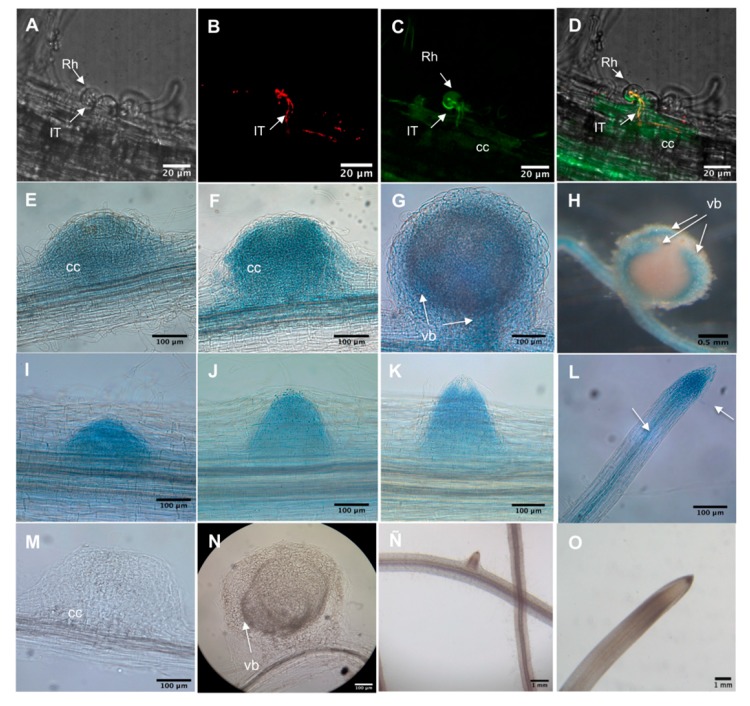
Spatiotemporal activity of the *Phaseolus vulgaris–rhizobia symbiotic interaction (PvADFE)* promoter in *P. vulgaris* transgenic roots during nodulation. Promoter activity was observed in transgenic roots expressing 1.3 kb of the *PvADFE* promoter region fused to β-glucuronidase (GUS) activity and green fluorescent protein (GFP) (p*Pv*ADFE:GUS-GFP). (**A**–**D**) Activity of the promoter observed by confocal microscopy in infected root hairs of transgenic roots inoculated with *R. tropici*–DsRed at 5 dpi. (**A**) Transmitted light image; (**B**) red fluorescence emitted by *R. tropici* CIAT899–DsRed; (**C**) p*Pv*ADFE:GUS-GFP expression; (**D**) overlay image. (**E**–**H**) Promoter activity detected by GUS staining in a nodule primordium, young nodule, and vascular bundles of a mature nodule at (**E**) 5, (**F**) 7, (**G**) 14, and (**H**) 21 dpi, respectively. (**I**–**L**) GUS staining during development of a lateral root: (**I**–**K**) root primordium, and (**L**) apical region and vascular tissue (indicted by arrows) of a fully developed lateral root. (**M**–**O**) GUS staining in control roots. (**M**) Nodule primordium, (**N**) mature nodule, (**O**) root vascular tissue, and (**P**) root apical region. cc, cortical cells; IT, infection thread; Rh, root hair; vb, vascular bundles.

*PvADFE* promoter activity was monitored by measuring β-glucuronidase (GUS) activity or green fluorescent protein (GFP) fluorescence in hairy roots from composite plants. We analyzed the activity of the *PvADFE* promoter in infected root hairs of transgenic roots inoculated with *R. tropici*–DsRed. Promoter activity was detected in root hairs harboring growing ITs and in adjacent cortical cells undergoing division (Figure 1A–D). Furthermore, GUS activity was observed in cortical cells undergoing the initial cell divisions that form the nodule primordium at 5, 7, and 14 dpi (Figure 1E–G), and was restricted to vascular bundles in mature nodules (Figure 1H). Promoter activity was also found at sites of lateral root primordia, and subsequently became confined to the apical region and vascular bundles of fully developed lateral roots (Figure 1I–L). These spatiotemporal expression patterns of the *PvADFE* promoter compared to control roots (Figure 1M–O) suggest that PvADFE participates in IT progression and nodule organogenesis, as well as lateral root development.

### 2.4. PvADFE Down-Regulation Increases the Number of Infection Events, Nodule Number, and Nitrogen Fixation in Rhizobium-Inoculated Transgenic Roots

To gain an insight into *PvADFE* gene function in symbiotic nodulation, its expression was altered by either RNAi knockdown or overexpression. The effectiveness and specificity of RNAi silencing were assessed by RT-qPCR expression analysis of *PvADF* genes in several independent transgenic roots. The *PvADFE* transcript level was 60% lower in RNAi transgenic roots than in control transgenic roots (Figure S6). Transcript levels of the other eight *PvADF* genes indicated that the *PvADFE*-RNAi construct specifically down-regulated *PvADFE* transcript in transgenic roots, validating the specificity of the construct designed (Figure S7).

**Figure 2:**
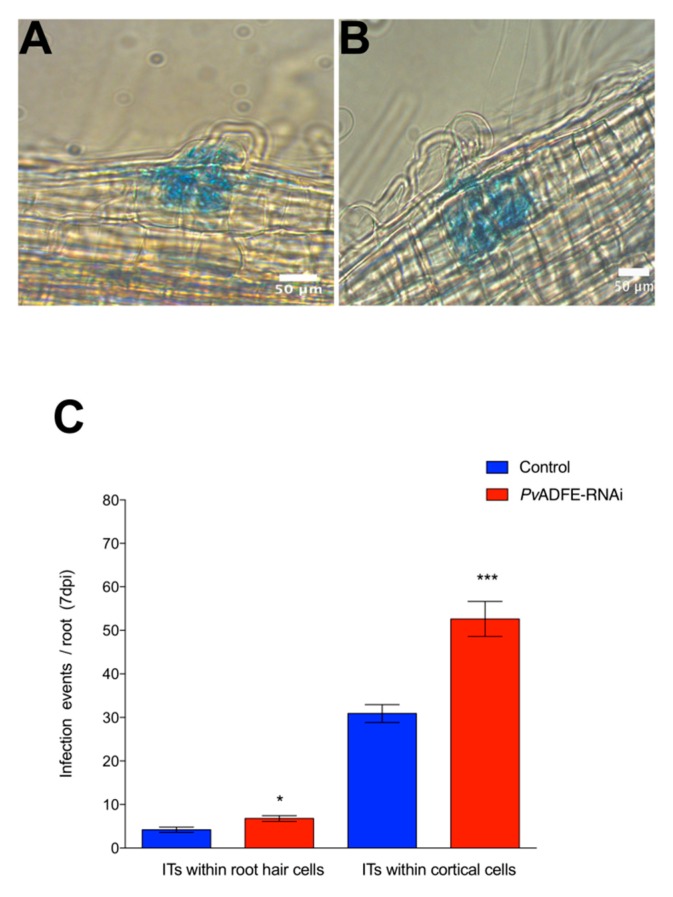
Analysis of infection events in root hairs of *PvADFE*-RNAi and control transgenic roots at 7 dpi with *R. tropici* expressing GUS. Micrographs show (**A**) branched infection threads (ITs) invading root cell layers in control transgenic roots and (**B**) ITs within the cortical cells in *PvADFE*-RNAi transgenic roots. (**C**) Number of infection events per root in *PvADFE*-RNAi and control transgenic roots at 7 dpi. Values are mean ± SEM with *n* > 9 roots per condition. * *p* < 0.05 and *** *p* < 0.005 according to Student’s *t*-test. Bars (**A**,**B**), 50 μm.

The participation of *PvADFE* in symbiosis was investigated in silenced inoculated roots and nodules. At 7 dpi, both *PvADFE*-RNAi and control transgenic roots showed ITs within cortical cells (Figure 2A and B, respectively); however, the number of infection events in *PvADFE*-RNAi transgenic roots was significantly higher than that in controls within root hair cells and cortical cells (Figure 2C). These results indicate that ITs in silenced roots can progress to characteristic nodule primordia that will develop determinate nodules.

**Figure 3:**
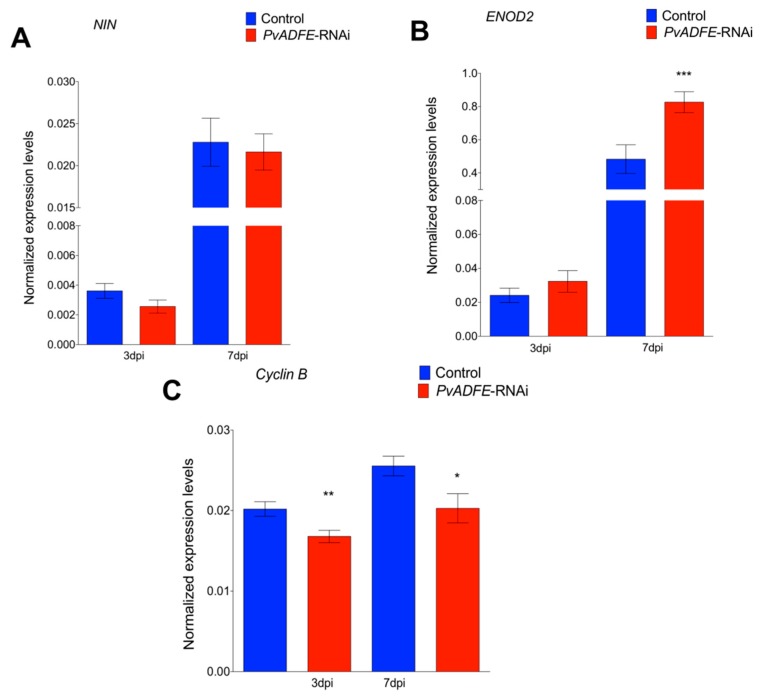
Reverse-transcription quantitative PCR analysis of early nodulins in *PvADFE*-RNAi and control transgenic roots after inoculation with *R. tropici* CIAT-899. (**A**) *NIN*, (**B**) *ENOD2*, and (**C**) *Cyclin B* transcript accumulation profiles determined by RT-qPCR of total RNA isolated from *PvADFE*-RNAi and control transgenic roots at 3 and 7 days after inoculation with rhizobia. Elongation factor *EF1*α was used as an endogenous reference gene for normalizing expression levels. Each bar represents mean ± SEM of two independent biological replicates with three technical repeats. * *p* < 0.05, ** *p* < 0.005, and *** *p* < 0.0001 based on Student’s *t*-test.

We next monitored the transcript accumulation profiles of *NIN* and *ENOD2*, which are associated with changes in nodulation signaling. Transcriptional activation of *NIN* [50] regulates the early steps of nodulation, such as NF-induced gene expression, IT formation, and initiation of nodule primordia [51]; hence, *NIN* is a specific marker for IT formation. *ENOD2* is induced during cortical cell division in the early phases of nodule development [52]. Even though we did not observe increased expression of *NIN* at 7 dpi (Figure 3A), a significantly increased transcript abundance of *ENOD2* in *PvADFE*-RNAi transgenic roots relative to controls was found (Figure 3B). Cyclin genes are suitable markers of dividing cells and are used in studies of plant developmental processes, such as nodule formation [53]. The B-type cyclins are highly expressed in meristem [54]. For this reason, we also analyzed *cyclin B* transcript levels at the same time points, and observed that the down-regulation of *PvADFE* significantly reduced *cyclin B* gene expression at 3 and 7 dpi (Figure 3C).

**Figure 4:**
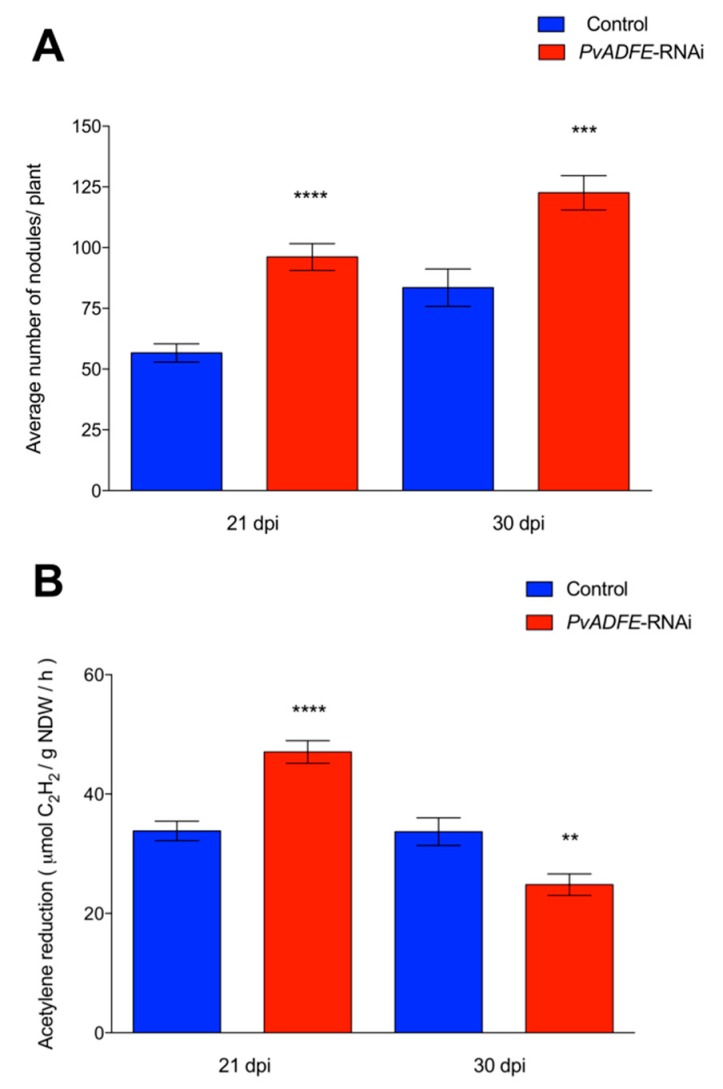
Nodulation and nitrogen fixation capacity of *PvADFE*-RNAi and control transgenic roots. (**A**) Nodules were collected and counted from control and *PvADFE*-RNAi transgenic roots inoculated with *R. tropici* CIAT-899 at 21 and 30 dpi. (**B**) Nitrogenase activity determined by acetylene reduction in control and *PvADFE*-RNAi nodules of transgenic roots at 21 and 30 dpi. NDW: Nodule dry weight. Bars represent mean ± SEM for three biological replicates with *n* > 7. ** *p* < 0.005, *** *p* < 0.0005, and **** *p* < 0.0001 based on Student’s *t*-test.

*PvADFE*-RNAi transgenic roots had 55 and 33% more nodules at 21 and 30 dpi than was observed in the control, respectively (Figure 4A). Interestingly, the overall distribution of nodule size and average nodule diameter on silenced roots was similar to that on control roots at all time points examined (Figure S8A,B). Furthermore, *PvADFE*-RNAi nodules at 21 dpi were 55% more effective in nitrogen fixation than control nodules, whereas the nitrogen-fixing ability of the *PvADFE*-RNAi nodules was 28% lower than that of controls at 30 dpi (Figure 4B). Altogether, our data point toward a fine-tuning role of the nodulation process mediated by PvADFE.

### 2.5. IT Progression, Nodule Number, and Nitrogen Fixation Are Impaired in PvADFE-Overexpressing Hairy Roots

**Figure 5:**
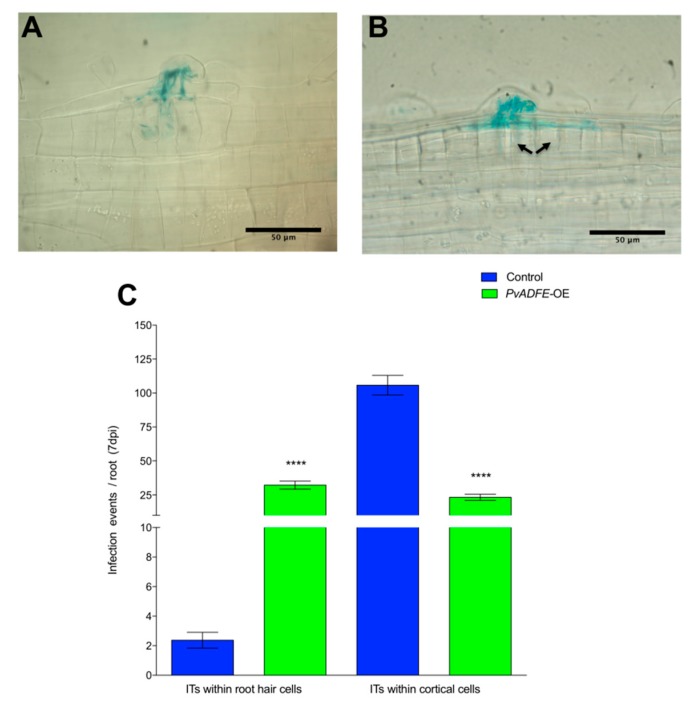
Infection events in root hairs of *PvADFE*-OE and control transgenic roots at 7 dpi with *R. tropici* expressing GUS. Micrographs show (**A**) branched ITs invading root cell layers in control transgenic roots and (**B**) ITs arrested within the epidermal cells in *PvADFE*-OE transgenic roots. Cortical cell division is indicated by black arrows. (**C**) Number of infection events per root in *PvADFE*-OE and control transgenic roots at 7 dpi. Values are mean ± SEM with *n* > 9 roots per each condition. **** *p* < 0.0001 according to Student’s *t*-test. Bars (**A**,**B**), 50 μm.

To further evaluate the function of ADF in inoculated roots and nodules, we overexpressed *PvADFE* (using the 35S promoter) in *P. vulgaris* transgenic roots. The *PvADFE* transcript level was 32-fold higher (*p* < 0.001, Student’s *t*-test) than in control transgenic roots, which contained an empty pH7WG2tdT vector (Figure S9). Contrary to the *PvADFE*-RNAi results, ITs were arrested at the base of the root hairs of *PvADFE*-overexpressing (*PvADFE*-OE) transgenic roots, although cell division was still observed in the outer cortex (Figure 5A,B, respectively), which can eventually develop into nodule primordia. The number of infection events in *PvADFE*-OE transgenic roots was significantly smaller than that in control roots. The 58% of the ITs were confined within the root hairs whereas 97% of ITs reached the cortical cells in control roots (Figure 5C). These results reinforce those obtained by *PvADFE*-RNAi, suggesting a role of PvADFE in fine-tuning nodulation.

**Figure 6:**
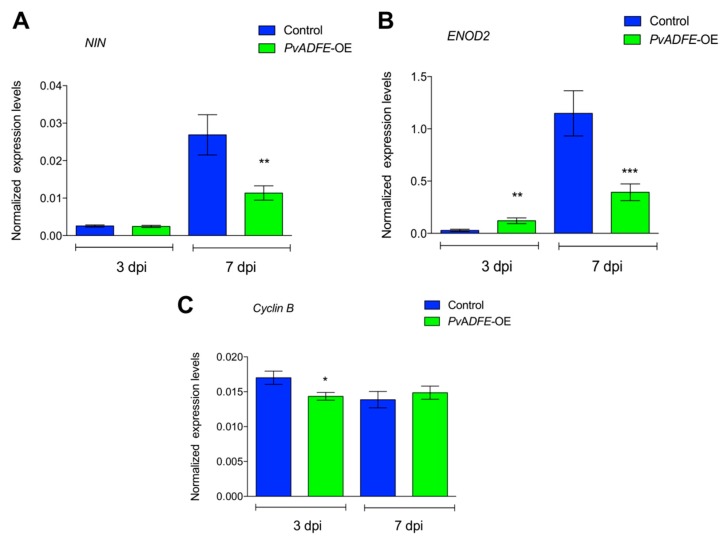
Reverse-transcription quantitative PCR analysis of early nodulins in *PvADFE*-OE and control transgenic roots after inoculation with *R. tropici* CIAT-899. (**A**) *NIN*, (**B**) *ENOD2*, and (**C**) *Cyclin B* transcript accumulation profiles determined by qRT-PCR of total RNA isolated from *PvADFE*-OE and control transgenic roots at 3 and 7 days after inoculation with rhizobia. Elongation factor *EF1α* was used as an endogenous reference gene for normalizing expression levels. Each bar represents mean ± SEM for two independent biological replicates with three technical repeats. * *p* < 0.05, ** *p* < 0.005, and *** *p* < 0.001 according to Student’s *t*-test.

Next, the transcript accumulation profiles of *NIN* and *ENOD2* genes were evaluated. Levels of *NIN* in *PvADFE*-OE transgenic roots were significantly lower (30-fold) than those in control roots at 7 dpi (Figure 6A). This down-regulated expression of *NIN* in *PvADFE*-OE transgenic roots was associated with a decreased number of ITs in the cortical cells at 7 dpi (Figure 5C). *ENOD2* transcript level was significantly higher in *PvADFE*-OE transgenic roots at 3 dpi compared with control roots; however, *ENOD2* transcript accumulation was significantly lower in the *PvADFE*-OE roots compared with the control transgenic roots at 7 dpi (Figure 6B). At 3 dpi, *cyclin B* transcript levels were slightly lower in *PvADFE*-OE transgenic roots compared with control roots, while only a slight difference was observed at 7 dpi in comparison with control transgenic roots (Figure 6C). The low expression levels of *NIN* and *ENOD2* at 7 dpi could explain the inefficiency of IT formation and progression, as well as the reduced number of nodules on *PvADFE*-OE roots; however, the levels of *cyclin B* transcript in *PvADFE*-OE roots were only affected at 3 dpi. We therefore next addressed whether this also affected cell division, primordia, and nodule development.

**Figure 7:**
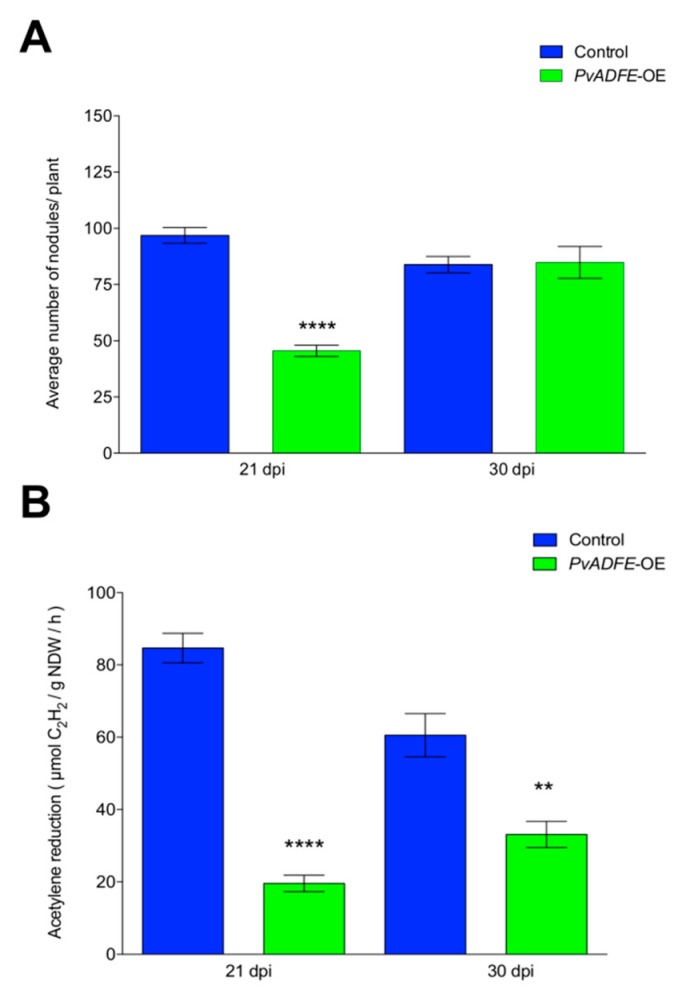
Nodulation and nitrogen fixation capacity of *PvADFE*-OE and control transgenic roots. (**A**) Nodules were collected and counted from control and *PvADFE*-OE transgenic roots inoculated with *R. tropici* CIAT-899 at 21 and 30 dpi. (**B**) Nitrogenase activity determined by the acetylene reduction assay in control and *PvADFE*-OE nodules of transgenic roots at 21 and 30 dpi. NDW: Nodule dry weight. Bars represent mean ± SEM for three biological replicates with *n* > 7. ** *p* < 0.005 and **** *p* < 0.0001 according to Student’s *t*-test.

Overexpression of *PvADFE* resulted in a 45% reduction in nodule number at 21 dpi in comparison to control roots. However, no changes in nodule number were observed at 30 dpi (Figure 7A). Although 50% less of ITs reach the cortical cells in *PvADFE*-OE transgenic roots (Figure 5C); roots are continuously infected by rhizobia, which may explain the similar number of nodules in control and *PvADFE*-OE transgenic roots at 30 dpi (Figure 7A). Based on an acetylene reduction assay, nodules from *PvADFE*-OE transgenic roots had 75% less nitrogen-fixing ability than control roots at 21 dpi and 50% less at 30 dpi (Figure 7B). We also found that the diameter and distribution of nodule size differed in *PvADFE*-OE and control transgenic roots at 7, 14, 21, and 30 dpi (Figure S10). Specifically, at all time points, *PvADFE*-OE had a higher proportion of small nodules (Group I, see Materials and Methods Section) compared to control transgenic roots (Figure S10A). In *PvADFE*-OE transgenic roots, the proportion of large nodules (Group III) at 30 dpi was low (24%), whereas this proportion was 39% in control roots. In addition, the average diameter of nodules in *PvADFE*-OE was smaller than that of nodules in control roots at the same stages (Figure S10B). These results indicate that overexpression or the general presence of *PvADFE* transcripts affects nodule development and function, confirming a role of *PvADFE* under silencing conditions.

## 3. Discussion

Plant *ADF* genes in angiosperms comprise a large family, which can be classified into four subclasses according to their tissue-specific expression and phylogeny. For example, Arabidopsis subclass I *ADFs*, *ADF1* to *ADF4*, are expressed strongly and constitutively in all vegetative tissues [35]. This ADF subclass has been shown to function in plant resistance to pathogenic microbes, fungi, and pests. Arabidopsis ADF4 functions as a susceptibility factor between the host plant and the powdery mildew fungus [55]. Control of *Myzus persicae* Sülzer infestation in *adf3* was restored by overexpression of the related *ADF4* or treatment with the actin cytoskeleton destabilizers cytochalasin D and latrunculin B [40]. Despite symbiotic and pathogenic interactions being different manifestations of the bacteria–host interaction, similar mechanisms exist in both processes to facilitate successful colonization. Here, we suggest that *PvADFE* participates fine-tuning the signaling pathway leading to the infection process and nodule organogenesis in *P. vulgaris* symbiosis.

In previous studies, we described early actin cytoskeleton rearrangements in bean root hair cells in response to specific NFs [3,18]. Actin dynamics are the result of highly regulated polymerization and depolymerization processes. A proteomics analysis conducted by our group showed that one of the *P. vulgaris* ADFs, PvADFE, increases 11.7-fold in relative abundance 30 min after rhizobial NF treatment. Herein, we establish that ADFE is required for both early and late symbiotic processes in bean.

Some reports have described actin cytoskeleton remodeling at the site of plant cell contact with pathogens [56,57,58,59]. Similar remodeling has been observed in symbioses; for instance, IT development requires the rearrangement of the actin cytoskeleton [60]. NF perception leads to fragmentation of longitudinal thick actin bundles, causing accumulation of finer and more diffuse actin at the root-hair tips [3,17]. The number of F-actin plus ends also increases, and F-actin is relocalized to the IT initiation sites [18]. ADFs are the first regulators of actin cytoskeleton organization and function in the disassembly of actin filaments [61]. We showed that the *PvADFE* promoter is activated in infected root hairs and in cortical cells during division (Figure 1A–D). Knockdown and overexpression of *PvADFE* had opposing effects on the number of infection events, nodule number, and nitrogen fixation. *PvADFE* silencing increased the number of infection events (Figure 2C), whereas *PvADFE* overexpression resulted in 58% of the ITs being confined within the root hairs (Figure 5C). Although the link between ADF and actin during symbiosis is unknown, cytoskeletal rearrangements have been linked to ADF after pathogen perception [37,55,62]. In *A. thaliana*, the *adf4* mutant fails to undergo actin rearrangements during innate immune signaling in response to treatment with a bacterial microbe-associated molecular pattern, elf26, but responds normally to a fungal microbe-associated molecular pattern, chitin [62]. Also, *AtADF4* is specifically required for the activation of *RPS5*-mediated resistance as well as MAPK signaling via the coordinated regulation of actin cytoskeletal dynamics and *R-gene* transcription [37]. The knockdown of *AtADF1* to *AtADF4* genes enhances resistance to the powdery mildew fungus *Golovinomyces orontii* [55], which is contrary to our results, suggesting a different role of ADF in symbiosis and fungal pathogen response. Then, we propose that cytoskeletal changes triggered by the depolymerization of F-actin during rhizobial invasion might be mediated by the participation of PvADFE, which may decrease the viscosity of the cytoplasm, and the relaxation of the cytoskeleton may facilitate successful infection, as previously suggested for nematode-infected roots [63]. However, in excess of ADF, this leads to actin filament stabilization and interferes with actin severing [64,65]. For instance, NtADF1 inhibits pollen tube growth in a dose-dependent manner, and very high levels of GFP-NtADF1 expression result in bundled or patchy regions of actin [44]. A similar scenario may occur in root hairs infected with rhizobia, in which PvADFE excess stabilizes actin filaments, blocking the required reorganization of actin in root hairs during IT initiation and IT progression, which has been widely reported [3,17]. It is possible that during rhizobial infection, ADF protein is accumulated; however, it might remain inactivated (e.g., by phosphorylation or pH) in order to mediate a successful rhizobial infection process.

The initiation of IT growth following NF perception in root hairs includes early changes in nodulin gene expression [9]. In the current study, we observed down-regulation of *NIN* and *ENOD2* in *PvADFE*-overexpressing transgenic roots at 7 dpi (Figure 6A,B), supporting the idea that *PvADFE* participates in nodulin signaling, in a similar manner to *AtADF4*, which is required for MAPK signaling in the presence of the bacterial effector AvrPphB [37]. Surprisingly, overexpression of *PvADFE* slightly affects the *cyclin B* expression at 3 dpi, suggesting that, at this stage, *PvADF* is involved in IT growth rather than primordia development. This role has also been suggested for *L. japonicus* Nap and Pir1, components of the Suppressor of cAMP receptor defect/WASP family verpolin homologous protein (SCAR/WAVE) complex, which induces actin nucleation and rearrangements via recruitment and activation of the Arp2/3 complex [15].

The silencing and overexpression of *PvADFE* also cause defects in nodule development. At 21 dpi, *PvADFE*-RNAi transgenic roots had 41% more nodules and these were 55% more effective in nitrogen fixation compared to the control (Figure 4), whereas 63% fewer nodules and a low level of nitrogen fixation were observed in transgenic roots overexpressing *PvADFE* (Figure 7). We revealed that the *PvADFE* promoter is also activated in the vascular bundles of mature nodules (Figure 1H). In this direction, gene expression was reported in vascular bundle tissue of petunia (*PhADF1*) and rice (*OsADF1* and *OsADF3*) [66,67]. Thus, overexpression of *PvADFE* might alter the actin filaments in vascular bundles of nodules, compromising the supply of nutrients and water transport to the nodule cells. In addition, nitrogen-fixing symbiosomes develop highly dynamic actin networks to support vesicle transport and promote growth [19,24]. Actin organization and remodeling associated with rhizobium release and symbiosome development can be described in three steps: (a) F-actin arrays channel elongating ITs and mediate the release of infection droplets, (b) a network of actin microfilaments embraces the developing symbiosomes, and (c) short F-actin fragments and actin dots align with the mature symbiosomes [19]. Therefore, the small nodules and low level of nitrogen fixation observed in transgenic roots overexpressing *PvADFE* suggest that an altered actin rearrangement within infected nodule cells impairs bacteroid functional maturation within symbiosomes. By contrast, as reported [19], actin cytoskeleton channel formation that facilitates the infection droplet release, and symbiosome development and maturation are processes that might be facilitated by *ADF* silencing.

This work presents evidence that *ADF* is fine-tuning the symbiotic interaction between legumes and rhizobia. ADFE affects root hair infection, nodule development and nodule fixation. We are currently analyzing *adfe* mutations affecting Ser-6 (phosphomimetic PvADFE), and colocalization with actin cytoskeleton to better understand the function of this ADF and the actin dynamics in the rhizobia–*P. vulgaris* symbiosis.

## 4. Materials and Methods

### 4.1. Plant Growth Conditions and Rhizobia Inoculation

Seed of *P. vulgaris* cv. Negro Jamapa (obtained from the local farmers’ markets, Cuernavaca, Mexico) were surface-sterilized and germinated at 28 °C for 2 days in the dark. At 2 days post-germination, the apex of root was sectioned from seedlings and frozen in liquid nitrogen. Separately, the root hairs were gently broken off from roots without apex using a magnetic stir bar in a stainless-steel tank with liquid nitrogen. Consequently, the tripped roots were separated by filtering through a colander, and the root hairs were collected in the liquid nitrogen remained. All the material was stored at −70 °C until subsequent RNA extraction and reverse-transcription quantitative PCR (RT-qPCR) assays.

Composite common bean plants were generated according to the protocol developed by Estrada-Navarrete et al. [68]. Hairy roots (10–13 days post-emergence) were generated using *Rhizobium rhizogenes* strain K599 [69]. Transgenic composite plants carrying the corresponding construct were observed under epifluorescence microscopy to confirm the presence of the reporter gene (GFP or DsRed), and untransformed roots were removed. Composite common bean plants were planted in pots with vermiculite and inoculated with 20 mL of *R. tropici*–GUS or *R. tropici*–DsRed suspension at an optical density at 600 nm (OD_600_) of 0.05 (undiluted suspension had OD_600_ = 0.8–1.0). Plants were grown under greenhouse conditions with a controlled environment (26–28 °C, 16 h light:8 h dark) and were watered with B&D medium [70].

### 4.2. Identification of PvADF Sequences, Three-Dimensional Structure Analysis, and Relative Transcript Accumulation in Different Organs and Tissues

Using the Phvul006G132700.1 sequence as template, searches for ADFs in the Phytozome v12 database (http://phytozome.jgi.doe.gov/pz/portal.html) were performed for *P. vulgaris*, *G. max*, *M. truncatula*, *A. thaliana*, *O. sativa*, and *Z. mays*. *L. japonicus,* and *V. unguiculata* protein sequences homologous to ADF were identified and downloaded from the LIS (http://legumeinfo.org) and CGKB (http://cowpeagenomics.med.virginia.edu/CGKB/) databases, respectively (Table S4). PvADF sequences were arbitrarily named from PvADFA to PvADFI based on homology with Arabidopsis ADFs. A phylogenetic tree was generated by the maximum likelihood method based on the JTT matrix and FreeRate with 3 categories using IQ-TREE software version 1.3.11.1 for Linux [71] from 10,000 bootstrap replicates [72]. Multiple sequence alignment of the ADF amino acid sequences was performed using MUSCLE software for Linux [73] and edited using the web service Gblocks 0.91b (http://phylogeny.lirmm.fr/phylo_cgi/one_task.cgi?task_type=gblocks). The generated data in Newick tree format were visualized and edited in MEGA X [74].

PvADFE was selected for further analysis. A three-dimensional model of PvADFE was constructed using the I-TASSER server, http://zhanglab.ccmb.med.umich.edu/I-TASSER/ [75], based on homology with the Arabidopsis ADF1 crystal structure, PDB accession number 1FS7 [48]. The model was analyzed using Protean 3D software (DNAStar).

The *P. vulgaris* Genome Expression Atlas (PvGEA, http://plantgrn.noble.org/PvGEA/) was used in order to estimate the *PvADF* transcript accumulation profile from different organs and tissues of *P. vulgaris* analyzed by RNA sequencing and expressed as reads per kilobase of transcript per million mapped reads.

### 4.3. RT-qPCR Assays

High quality RNA was isolated from frozen tissues using TRIzol® Reagent (Invitrogen, Life Technologies, USA) following the manufacturer’s recommendations. RNA integrity and concentrations were determined by electrophoresis and NanoDrop (2000c, Thermo Scientific) spectrophotometry, respectively. Genomic DNA contamination from RNA samples was removed by incubating the samples with RNase-free DNase (1 U·μL^−1^) at 37 °C for 15 min and then at 65 °C for 10 min. RT-qPCR assays were performed using an iScript^™^ One-step RT-PCR Kit with SYBR® Green (Bio-Rad) from DNA-free RNA samples, according the manufacturer’s recommendations, in a LightCycler® Nano cycler (Roche). RNA template concentration was 40 ng (10 ng·μL^−1^) in each reaction. DNA-free RNA samples were used as a control to confirm the absence of DNA contamination. Relative expression values were calculated using the formula 2^−ΔCT^, where cycle threshold value (ΔCt) is the cycle threshold (Ct) of the gene of interest minus the Ct of the reference gene [76]. RT-qPCR data were generated from at least two biological replicates with three independent plants each one, together with three technical replicates. The gene encoding elongation factor 1-alpha (*EF1α*) was used as a reference gene to normalize the experimental data. The genes analyzed are listed in Table S5, together with their specific oligonucleotides used.

### 4.4. Plasmid Construction

To generate the RNAi construct, a fragment corresponding to the 3′-untranslated region of *PvADFE* was amplified from cDNA isolated from common bean roots at 2 days post germination using the following primers: *Pv*ADFE-RNAi-Up (5′-GTACGCTTTCTGGTGGGAGCAC-3′) and *Pv*ADFE-RNAi-Lw (5′-ACAAAAGAAAGCATATATCGTCCAAA-3′). The resulting PCR product was cloned into pENTR/D-TOPO (Invitrogen) and transformed by heat-shock into *Escherichia coli* TOP10 chemically competent cells. The recombination into the destination vector pTdT-DC-RNAi [77] was performed with the LR clonase, using the Gateway System (Invitrogen). The appropriated orientation of the insert was confirmed by PCR and sequencing using the *Pv*ADFE-RNAi-Up primer for the pTdT-*Pv*ADFE-RNAi plasmid together with WRKY-5-Rev (5′-GCAGAGGAGGAGAAGCTTCTAG-3′) or WRKY-3-Fwd (5′-CTTCTCCAACCACAGGAATTCATC-3′) primer. As a control, a truncated and irrelevant sequence from *A. thaliana* pre-mir159 (kindly provided by Dr. José Luis Reyes), lacking the target sequence of miR159 (ACAGTTTGCTTATGTCGGATCCATAATATATTTGACAAGATACTTTGTTTTTCGATAGATCTTGATCTGACGATGGAAGTAGAGCTCTACATCCCGGGTCA), was cloned into the pTdT-DC-RNAi vector. The correct orientation of the sequence in the construct was confirmed by DNA sequencing.

To construct an overexpression vector for *PvADFE*, the 833-bp ORF of *PvADFE* (Phvul006G132700.1) including the 5′-untranslated region (154 bp) and a 3’-untranslated (169 bp) fragment was isolated from *P. vulgaris* cDNA. This region was amplified from *P. vulgaris* root cDNA at 2 days post-germination using *PvADFE*-OE-Up and *PvADFE*-OE-Lw primers (Table S5). The fragment was cloned into the pENTR/D-TOPO vector (Invitrogen) and sequenced. The resulting pENTR-*PvADFE* plasmid was recombined into the binary vector pH7WG2tdT (constructed by Marco A. Juárez-Verdayes) under the control of the constitutive 35S promoter (Figure S11). Briefly, this vector was derived from vector pH7WG2D [78]; the cassette pEgfpER and the 35S terminator were respectively replaced by a TdTomato (red fluorescent protein) reporter obtained from the pTd-DC-RNAi vector [77] and an E9 terminator. Empty pH7WG2tdT vector was used as the control.

To develop a p*PvADFE*:GUS-GFP construct, a 1383-bp fragment upstream of the *PvADFE* translation start site was amplified using bean genomic DNA and primers p*Pv*ADFE-Up and p*Pv*ADFE-Lw (Table S5) and cloned into vector pENTR/SD/D-TOPO (Invitrogen). The Gateway LR reaction was performed between the entry vector pENTR/SD/D-TOPO-*Pv*ADFE and the destination vector pBGWFS7.0 [78], according to the manufacturers’ instructions (Invitrogen). Control transgenic roots harbored a cassette with no promoter sequences upstream of the GFP-GUS sequence.

### 4.5. Promoter Activity Analysis

Composite *P. vulgaris* plants harboring p*PvADFE*:GUS-GFP were transferred into pots of vermiculite and each plant was inoculated with 20 mL of *R. tropici*–DsRed suspension (with an OD_600_ of 0.05). Roots and nodules were collected at 5, 7, 14, and 21 dpi (days post-inoculation). Samples were histochemically analyzed for GUS activity according to the method of Jefferson [79] and images were acquired with a Retiga 4000R CCD camera coupled to a Nikon TE300 inverted microscope. Promoter activity during early infection events was detected using an inverted confocal microscope (Nikon eclipse Ti in combination with a Yokogawa CSU-W1 spinning disk confocal system) and images were processed using ImageJ version 1.48 (US National Institutes of Health). GFP fluorescence was excited at 488 nm, while DsRed fluorescence was excited at 543 nm.

### 4.6. Analysis of Infection Events, Nodule Number, and Nodule Diameter

Transgenic roots expressing red fluorescent protein (TdTomato from vector pH7WG2tdT) were selected as described above. These roots were transferred to pots with vermiculite and inoculated with *R. tropici*–GUS to analyze IT progression, nodulation, and nitrogen fixation. Infection events were analyzed in the control and *PvADFE* transgenic roots under a Zeiss Axioskop light microscope (Carl Zeiss, Jena, Germany) equipped with a ×63 objective. Images were captured by a Nikon Coolpix 5000 camera with a UR-E6 adapter and an MDC lens attached to the microscope. GUS activity was analyzed according to the method of Jefferson [79]. Images of nodulated transgenic roots at 7, 14, 21, and 30 dpi stained with GUS were taken using a Perfection 4490 scanner (Epson) and captured in TIFF format at a resolution of 6108 × 6108 pixels. Nodule diameter was measured using ImageJ 1.48 (US National Institutes of Health) and classified according to their diameter (d) into four groups: Group I (d < 0.5 mm), Group II (0.5 < d ≤ 1.0 mm), Group III (1.0 < d ≤ 1.5 mm), and Group IV (1.5 < d < 2.0 mm). The number of nodules was counted manually at 21 and 30 dpi.

### 4.7. Acetylene Reduction Analysis

Acetylene reduction [80] was used to quantify the nitrogenase activity in transgenic nodules at 21 and 30 dpi. Nodulated plant roots were transferred to bottles with rubber seal stoppers by injecting acetylene to a final concentration of 10% of the gas phase. Each sample was incubated for 120 min at room temperature, and ethylene production was determined by gas chromatography in a Variant model 3300 chromatograph. Specific activity is expressed as μmol ethylene^−1^·(g nodule dry weight)^−1^·h^−1^.

### 4.8. Statistical Analysis

Statistical analyses were computed using GraphPad Prism version 6.00 for Windows, (GraphPad Software, San Diego, CA, USA). Significance tests were performed using an unpaired Student’s *t*-test. Differences were considered significant if *p* < 0.05. Results are presented as means ± standard error of the mean.

## 5. Conclusions

In this study, we demonstrated that *ADFE* is preferentially expressed in rhizobia-inoculated roots and nodules. Functional characterization showed that ADF overexpression and silencing affect infection threads number, nodule number, average nodule diameter, and nitrogen fixation. Altogether, our results revealed that ADFE is fine-tuning the symbiotic interaction between common bean and rhizobia.

## Supplementary Materials

Supplementary materials can be found at https://www.mdpi.com/1422-0067/21/6/1970/s1. Figure S1. Exon–intron organization of *ADF* genes; Figure S2. Protein structure of *P. vulgaris* actin depolymerizing factors (PvADFs); Figure S3. Phylogenetic tree of ADF family proteins; Figure S4. Expression profile of *P. vulgaris ADF* genes in root hairs, apices, and stripped roots from seedlings harvested at 2 days post-germination; Figure S5. Transcript abundance of *ADF* genes in different organs and tissues of *P. vulgaris*; Figure S6. Reverse-transcription quantitative PCR analysis of *PvADFE* silencing in composite common beans roots; Figure S7. Expression of *PvADF* genes in control and *PvADFE*-RNAi transgenic roots at 10 days post emergence; Figure S8. Nodule diameters on *PvADFE*-RNAi and control transgenic roots after inoculation with *R. tropici* expressing GUS; Figure S9. Reverse-transcription quantitative PCR analysis of *PvADFE* overexpression in composite common bean roots; Figure S10. Nodule diameters on *PvADFE*-OE and control transgenic roots after inoculation with *R. tropici* expressing GUS; Figure S11. In silico map of the pH7WG2tdT vector; Table S1. Size of the *ADF* gene family in various plants; Table S2. Percentage of nucleotide sequence identity among *P. vulgaris ADF* genes; Table S3. Percentage of amino acid sequence identity between PvADF and AthADF proteins; Table S4. Annotation of ADFs aminoacid sequences used for the phylogenetic analysis; Table S5. Gene specific oligonucleotides used.

## Abbreviations

ADFs	Actin Depolymerization Factors
NFs	Nod factors
IT	Infection Thread
ARP3	Actin Related Protein 3
SCARN	Suppressor of cAMP Receptor Refect-Nodulation
ABP	Actin-Binding
ADF-H	Proteins Actin-Depolymerizing Factor Homology
PIP_2_	Phosphatidylinositol 4,5-bisphosphate
RPS5	Disease Resistance Protein 5
dpi	days post-inoculation
GFP	Green Fluorescent Protein
NDW	Nodule Dry Weight
GUS	β-glucuronidase
NIN	Nodule Inception
ENOD2	Early Nodulin2
