# Supplementary material for: Actin Depolymerizing Factor Modulates Rhizobial Infection and Nodule Organogenesis in Common Bean
Source: Int J Mol Sci. 2020 Mar 13;21(6):1970. doi: 10.3390/ijms21061970 (PMC7139724; doi:10.3390/ijms21061970)
Supplement: Supplementary file 1 [file ijms-21-01970-s001.pdf]

Supplementary Material

Actin Depolymerizing Factor Modulates Rhizobial Infection and Nodule Organogenesis in Common Bean

Yolanda Ortega-Ortega, Janet Carrasco-Castilla, Marco A. Juárez-Verdayes, Roberto Toscano-Morales, Citlali Fonseca-García, Noreide Nava, Luis Cárdenas and Carmen Quinto

Supplementary Figures

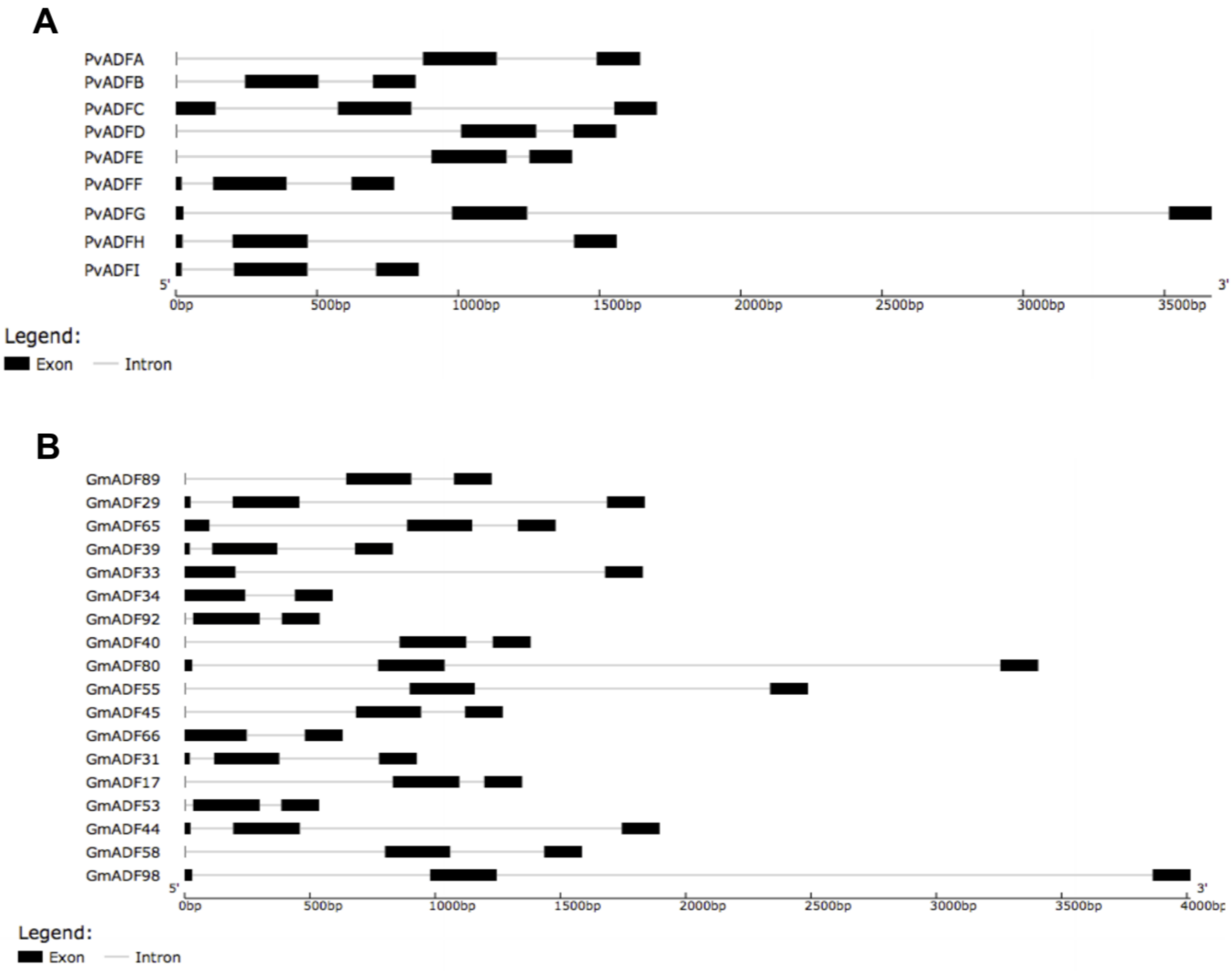

Figure S1. Exon–intron organization of *ADF* genes. Schematic representation of the gene structures of **(A)** *P. vulgaris ADF*s and **(B)** *G. max ADF*s generated using the Gene Structure Display Server tool (http://gsds.cbi.pku.edu.cn). Exons are indicated by black boxes and introns by gray lines.

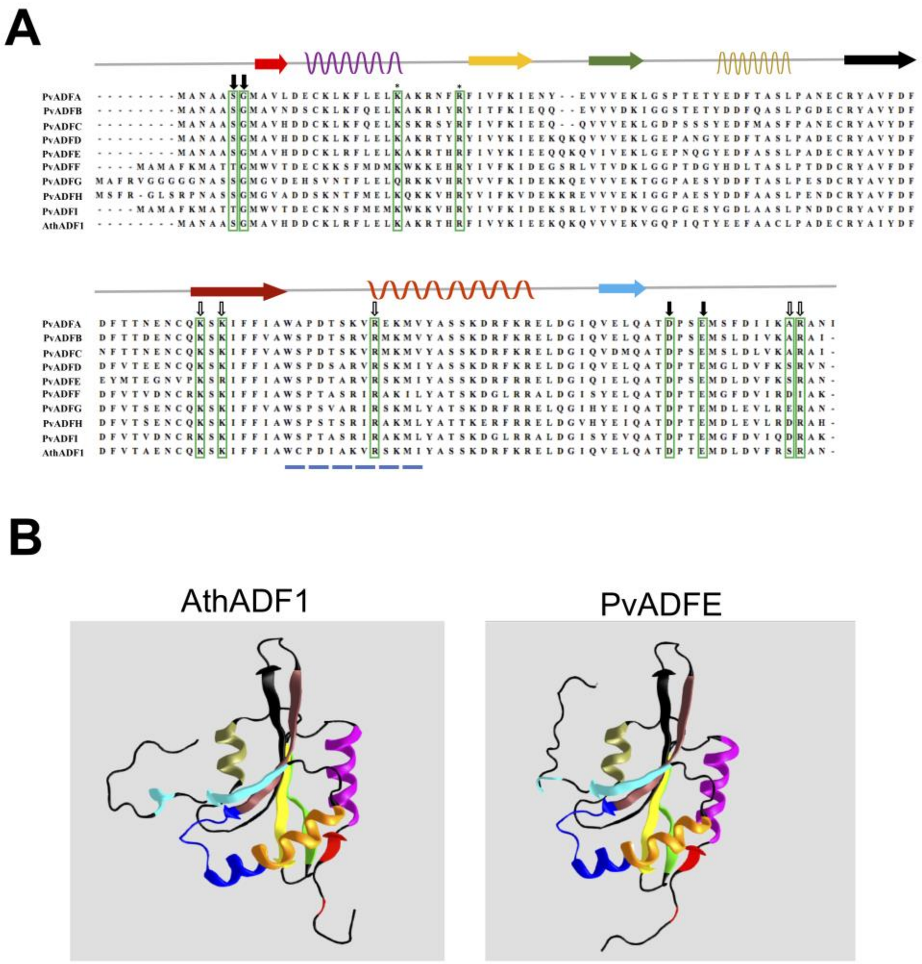

Figure S2. Protein structure of *P. vulgaris* actin depolymerizing factors (PvADFs). **(A)** Alignment of the deduced amino acid sequences of PvADF and AthADF1. The green boxed area indicates several conserved residues: nuclear localization signal is indicated by asterisks; white and black arrows indicate the binding sites of F-actin and G-actin, respectively. Blue dashed underlining represents PIP_2/actin binding. Conserved predicted secondary structures (wavy lines for α-helices and arrows for β-sheets) are shown above the sequences in colors corresponding to those used in the three-dimensional models. (**B)** Crystal structure of AthADF1 (1F7S) (on the left) [46], and predicted three-dimensional structure of PvADFE (on the right) compared by the DNAStar Protean 3D program.

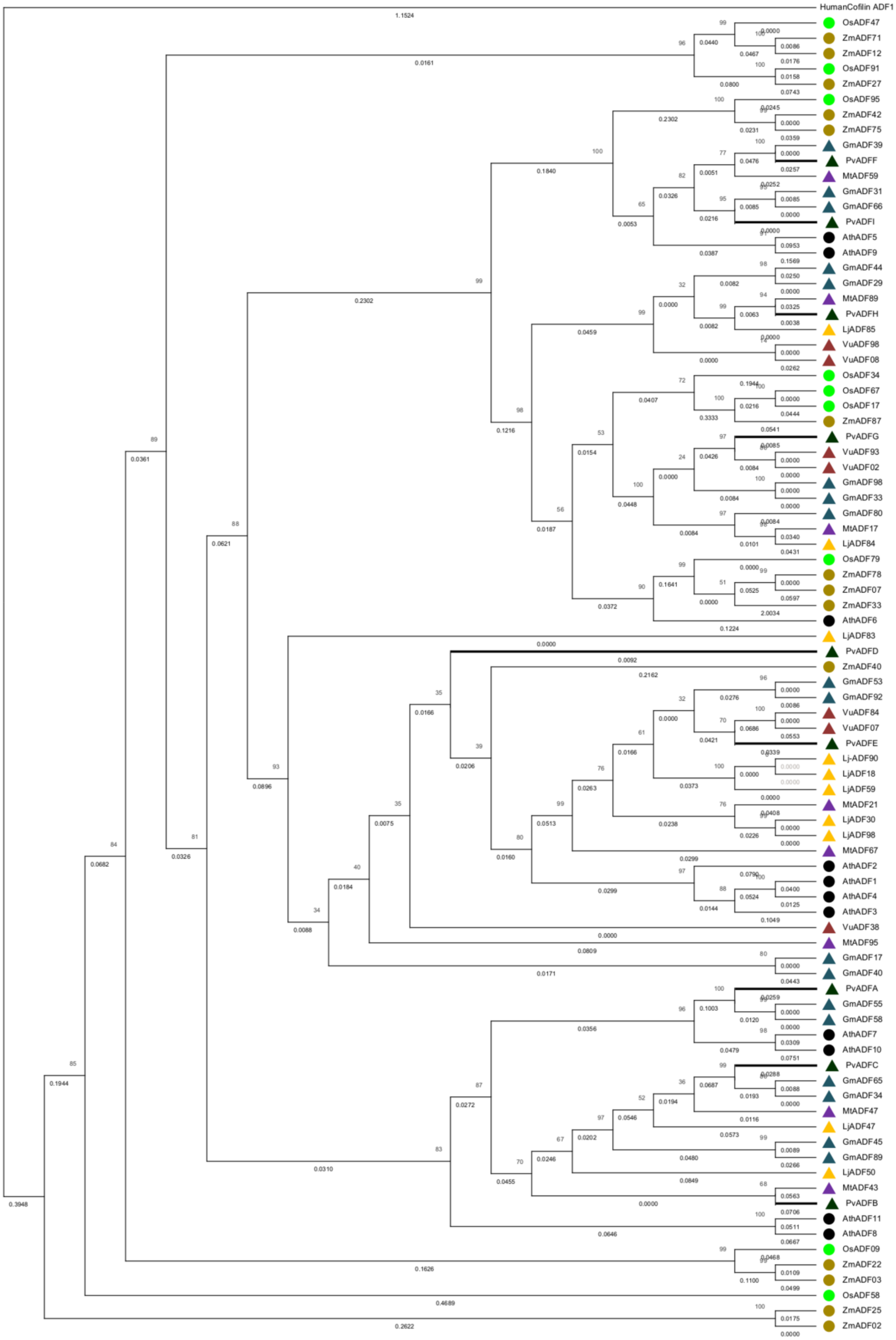
HumanCofilin ADF1
OsADF47
ZmADF71
ZmADF12
OsADF91
ZmADF27
OsADF95
ZmADF42
ZmADF75
GmADF39
PvADFF
MtADF59
GmADF31
GmADF66
PvADFI
AthADF5
AthADF9
GmADF44
GmADF29
MtADF89
PvADFH
LjADF85
VuADF98
VuADF08
OsADF34
OsADF67
OsADF17
ZmADF87
PvADFG
VuADF93
VuADF02
GmADF98
GmADF33
GmADF80
MtADF17
LjADF84
OsADF79
ZmADF78
ZmADF07
ZmADF33
AthADF6
LjADF83
PvADFD
ZmADF40
GmADF53
GmADF92
VuADF84
VuADF07
PvADFE
Lj-ADF90
LjADF18
LjADF59
MtADF21
LjADF30
LjADF98
MtADF67
AthADF2
AthADF1
AthADF4
AthADF3
VuADF38
MtADF95
GmADF17
GmADF40
PvADFA
GmADF55
GmADF58
AthADF7
AthADF10
PvADFC
GmADF65
GmADF34
MtADF47
LjADF47
GmADF45
GmADF89
LjADF50
MtADF43
PvADFB
AthADF11
AthADF8
OsADF09
ZmADF22
ZmADF03
OsADF58
ZmADF25
ZmADF02

Figure S3. Phylogenetic tree of ADF family proteins. Phylogenetic assembly of the ADF sequences from *Glycine max* (given in tree as Glyma), *Lotus japonicus (*Lj*)*, *Vigna unguiculata* (Vu), *Medicago truncatula* (Mt), *Zea mays* (Zm), *Oryza sativa* (Os), and *Arabidopsis thaliana* (Ath), labelled with circles and triangles in different colors. PvADFs for expression analysis are marked by black triangles. The human cofilin/ADF1 sequence was included as the root. Bootstrap values (as percentages of 10 000 replicates) are shown at nodes.

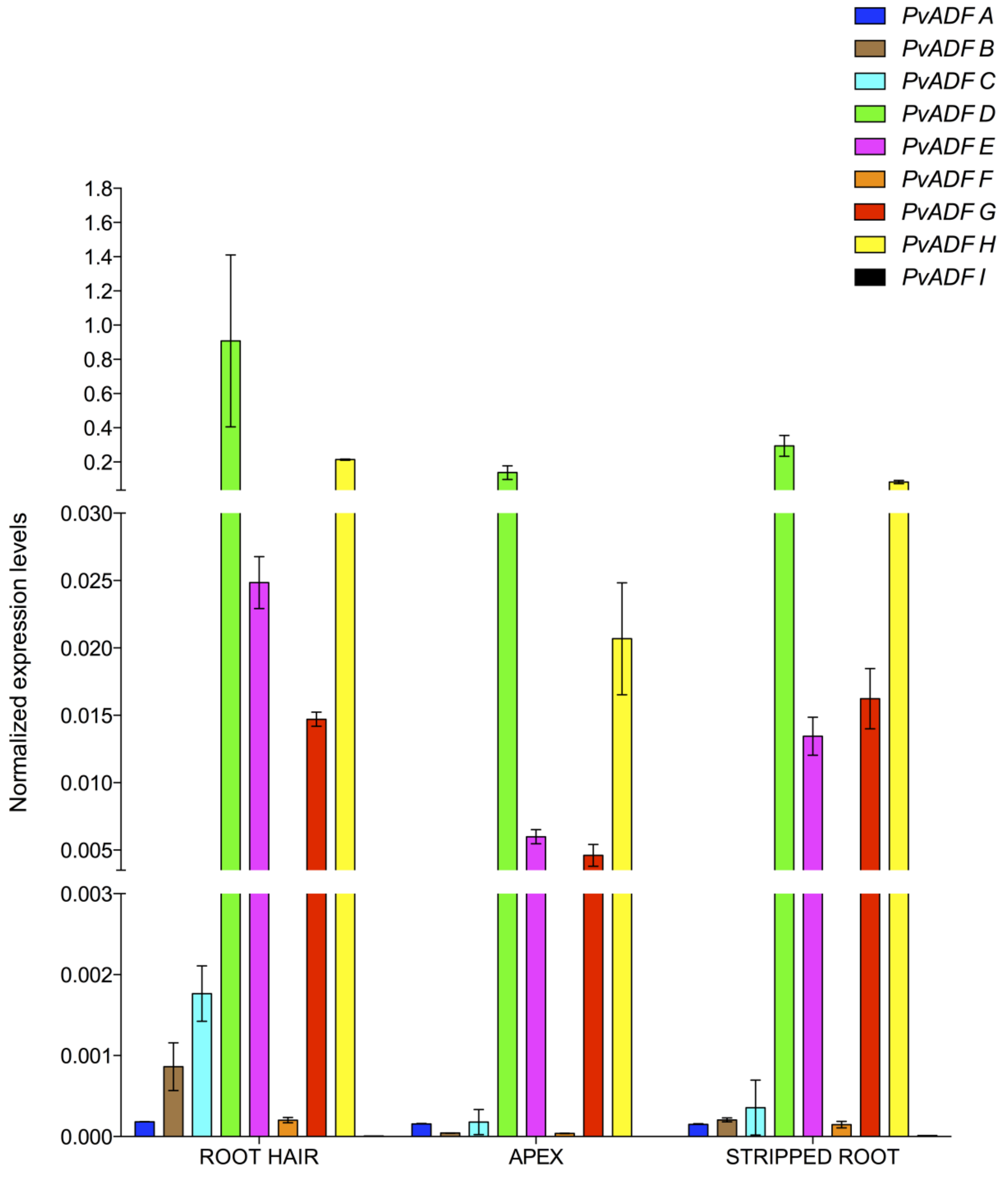

Figure S4. Expression profile of *P. vulgaris ADF* genes in root hairs, apices, and stripped roots from seedlings harvested at 2 days post-germination. Bars represent means ± SEM for three biological replicates with three technical repeats each. Elongation factor *EF1α* was used as the endogenous reference gene.

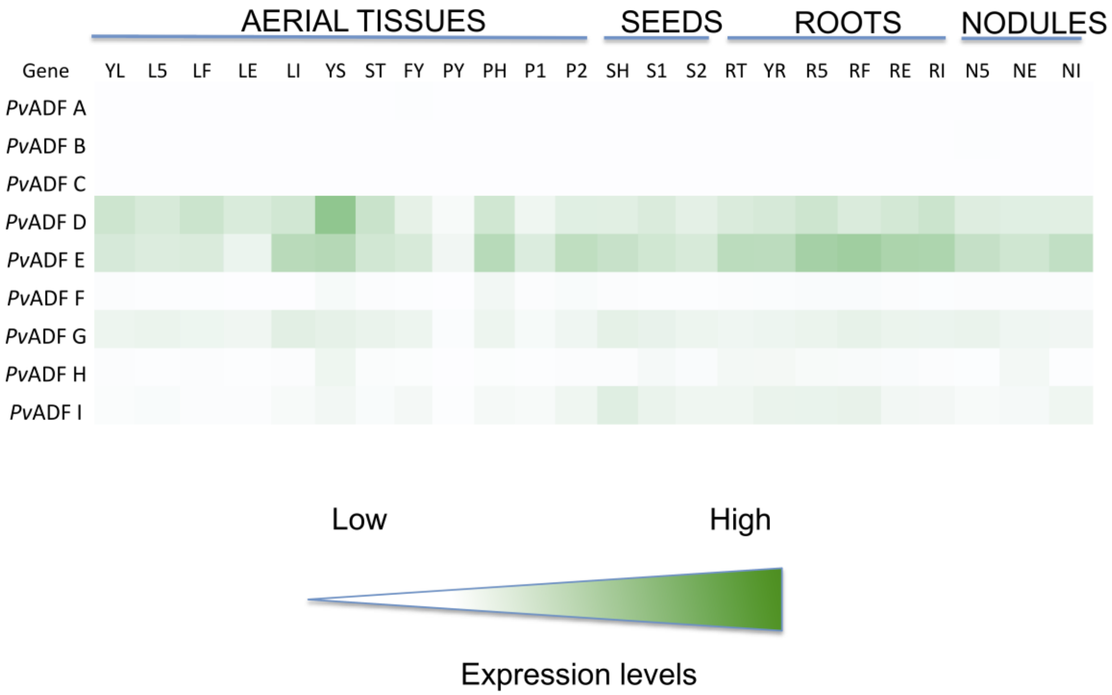

Figure S5. Transcript abundance of *ADF* genes in different organs and tissues of *P. vulgaris*. Heat map expression profiles highlighting the most abundant *PvADF* transcripts in organs and tissues of *P. vulgaris*. *PvADF* expression profile in inoculated roots and nodules. Expression was analyzed using the *Phaseolus vulgaris* Gene Expression Atlas (PvGEA): http://plantgrn.noble.org/PvGEA/. **YL**, fully expanded 2nd trifoliate leaf tissue from fertilized plants; **L5**, leaf tissue collected 5 days after plants were inoculated with effective rhizobium; **LF**, leaf tissue from fertilized plants collected at the same time as LE and LI; **LE**, leaf tissue collected 21 days after plants were inoculated with effective rhizobium; **LI**, leaf tissue collected 21 days after plants were inoculated with ineffective rhizobium; **YS**, all stem internodes above the cotyledon collected at the 2nd trifoliate stage; **ST**, shoot tip, including

the apical meristem, collected at the 2nd trifoliate stage; **FY**, young flowers, collected prior to floral emergence; **PY**, young pods, collected 1 to 4 days after floral senescence, containing developing embryos at the globular stage; **PH**, pods approximately 9 cm long, associated with seeds at the heart stage (pod only); **P1**, pods between 10 and 11 cm long, associated with stage 1 seeds (pod only); **P2**, pods between 12 and 13 cm long, associated with stage 2 seeds (pod only); **SH**, heart stage seeds, between 3 and 4 mm across and approximately 7 mg; **S1**, stage 1 seeds, between 6 and 7 mm across and approximately 50 mg; **S2**, stage 2 seeds, between 8 and 10 mm across and between 140 and 150 mg; **RT**, root tips, 0.5 cm of tissue, collected from fertilized plants at 2nd trifoliate stage of development; **YR**, whole roots, including root tips, collected at the 2nd trifoliate stage of development; **R5**, whole roots separated from 5-day-old pre-fixing nodules; **RF**, whole roots from fertilized plants collected at 21 dpi; **RE**, whole roots separated from fixing-positive nodules collected at 21 dpi; **RI**, whole roots separated from fixing-negative nodules collected at 21 dpi; **N5**, pre-fixing (effective) nodules collected at 5 dpi; **NE**, effectively fixing nodules collected at 21 dpi; **NI**, ineffectively fixing nodules collected at 21 dpi.

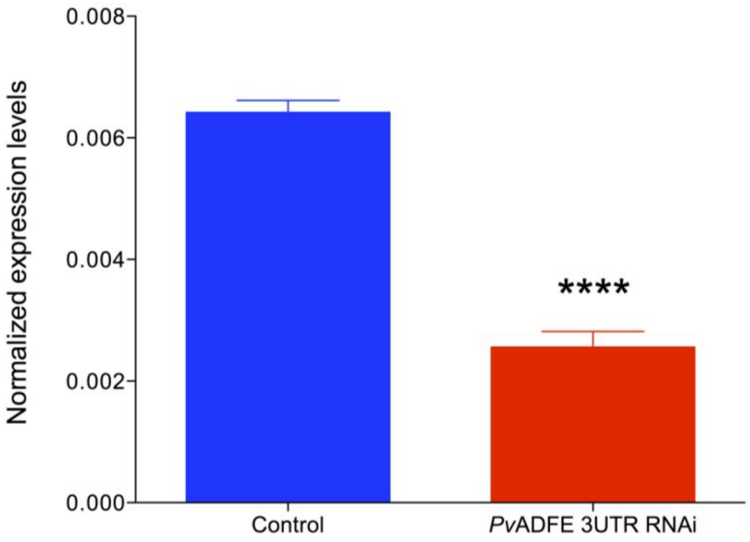

Figure S6. Reverse-transcription quantitative PCR analysis of *PvADFE* silencing in composite common bean roots. Transcript abundance was analyzed by RT-qPCR in transgenic roots transformed with empty vector or the *PvADFE*-RNAi construct. Elongation factor *EF1α* was used as an endogenous reference gene for normalizing expression levels. Bars represent mean ± SEM of two biological replicates with $n>4$. ****$p<0.0001$ based on Student's *t*-test.

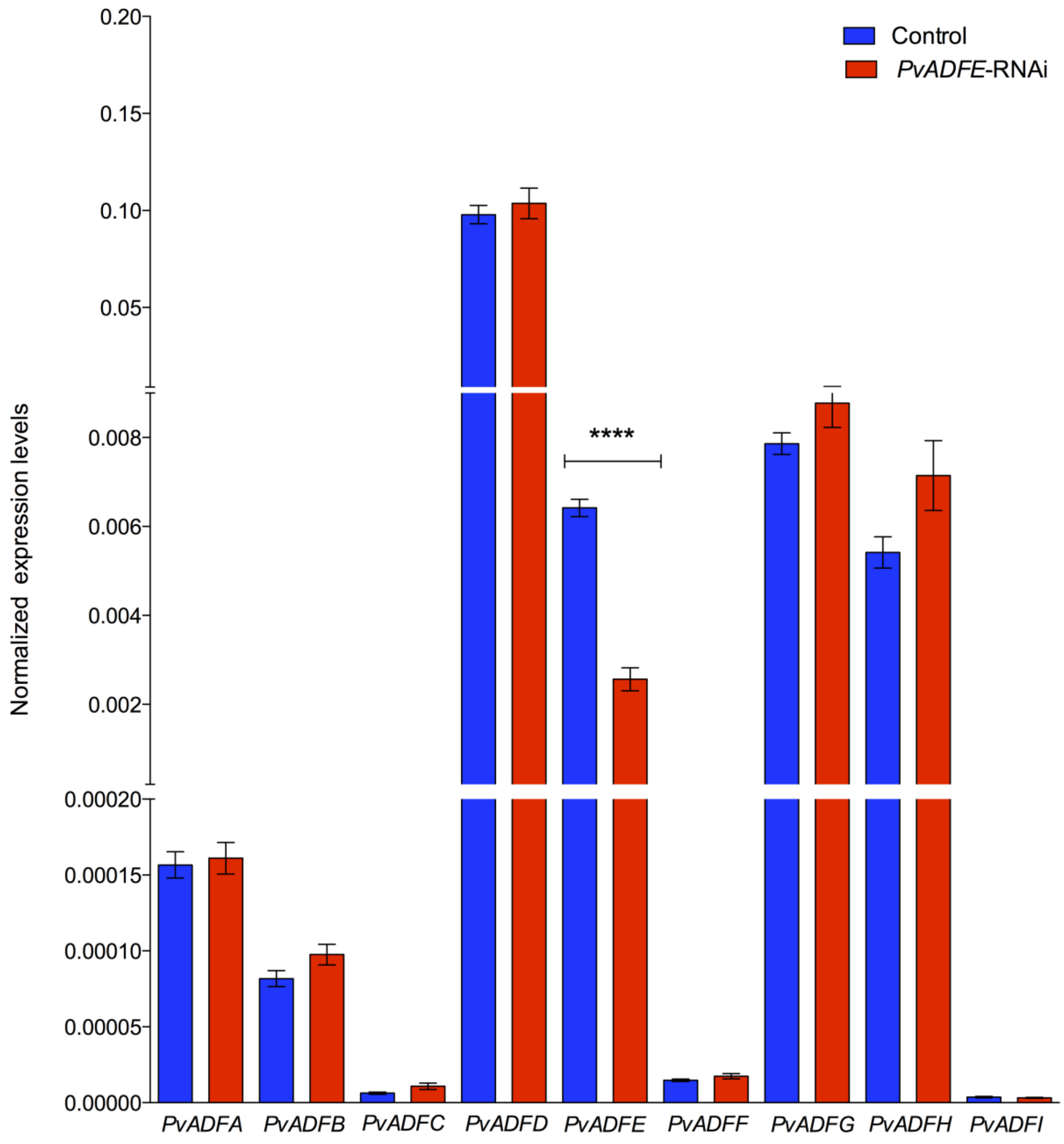

Figure S7. Expression of *PvADF* genes in control and *PvADFE*-RNAi transgenic roots at 10 days post emergence. Elongation factor *EF1α* was used as an endogenous reference gene for normalizing expression levels. Bars represent mean ± SEM for two biological replicates and three technical replicates. ****p<0.0001 determined using Student's t-test

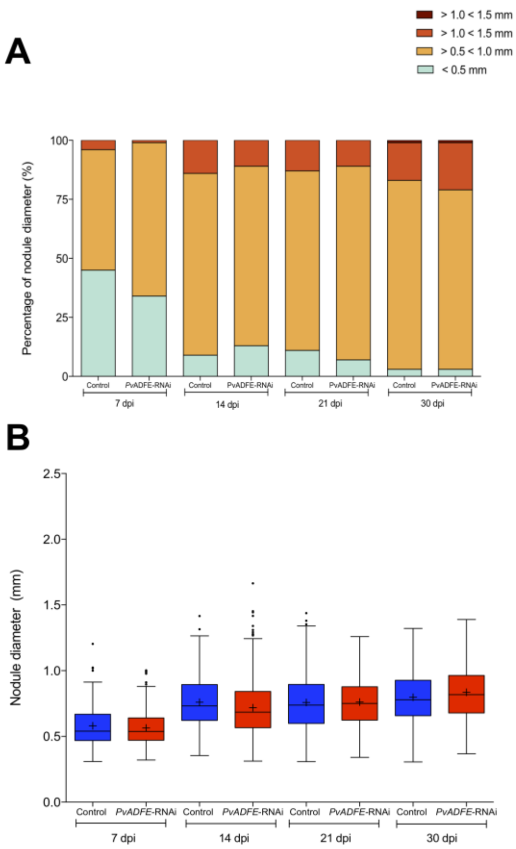

Figure S8. Nodule diameters on *PvADFE*-RNAi and control transgenic roots after inoculation with *R. tropici* expressing GUS. Nodules were collected at the indicated times and classified according to their diameter (d) into four groups: Group I (d<0.5 mm), Group II (0.5<d≤1.0 mm), Group III (1.0<d≤1.5 mm), and Group IV (1.5<d<2.0 mm). **(A)** Percentage of nodules having each diameter range and **(B)** distribution of nodule diameter on *PvADFE*-RNAi and control transgenic roots inoculated with the *R. tropici*–GUS strain. Center lines show medians; crosses indicate means; box limits indicate the first and fourth quartiles; whiskers extend 1.5 times the interquartile range from the first and third quartiles; outliers are represented by dots. *n*>394, from two individual biological replicates with five plants.

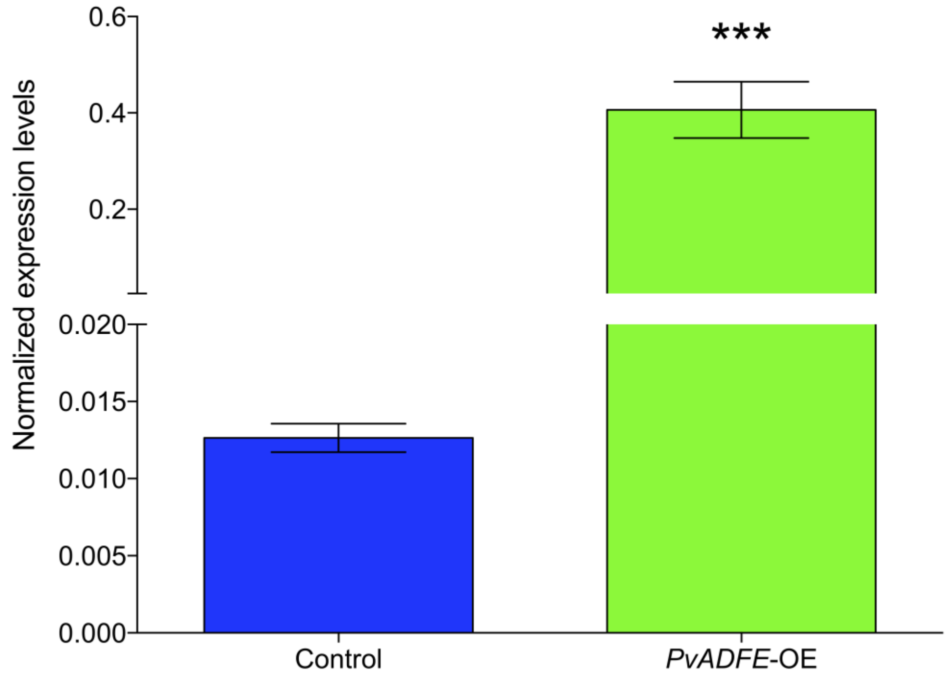

Figure S9. Reverse-transcription quantitative PCR analysis of *PvADFE* overexpression in composite common bean roots. Transcript abundance was analyzed by RT-qPCR in transgenic roots transformed with empty vector or the overexpression (*PvADFE*-OE) construct. Elongation factor *EF1α* was used as an endogenous reference gene for normalizing expression levels. Bars represent mean ± SEM for two biological replicates with $n>4$. ***$p<0.001$ based on Student's *t*-test.

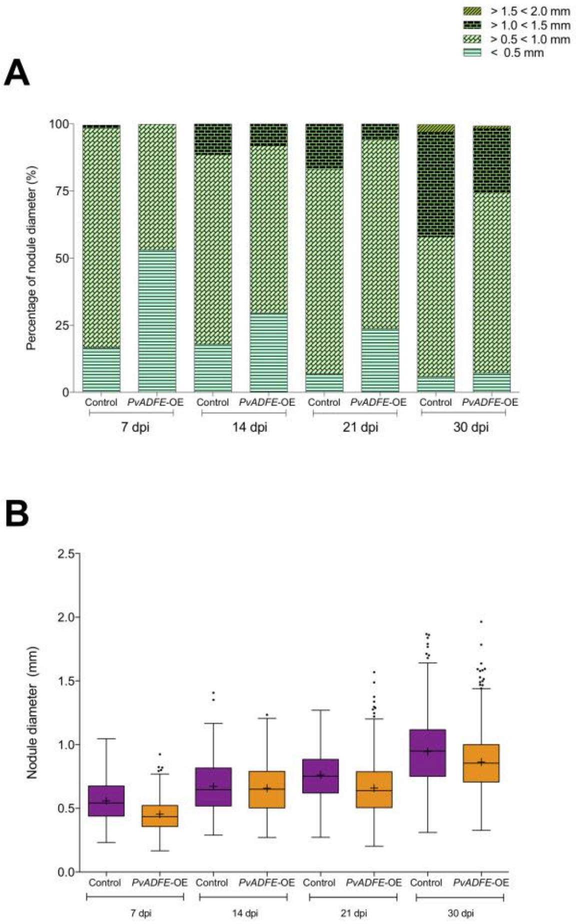

Figure S10. Nodule diameters on *PvADFE*-OE and control transgenic roots after inoculation with *R. tropici* expressing GUS. Nodules were collected at the indicated times and classified according to their diameter (d) into four groups: Group I (d<0.5 mm), Group II (0.5<d≤1.0 mm), Group III (1.0<d≤1.5 mm), and Group IV (1.5<d<2.0 mm). **(A)** Percentage of nodules having each diameter range and **(B)** distribution of nodule diameter on *PvADFE*-OE and control transgenic roots inoculated with the *R. tropici*–GUS strain. Center lines show the medians; crosses indicate means; box limits indicate the first and fourth quartiles; whiskers extend 1.5 times the interquartile range from the first and third quartiles; outliers are represented by dots. *n*>394, from two individual biological replicates with five plants.

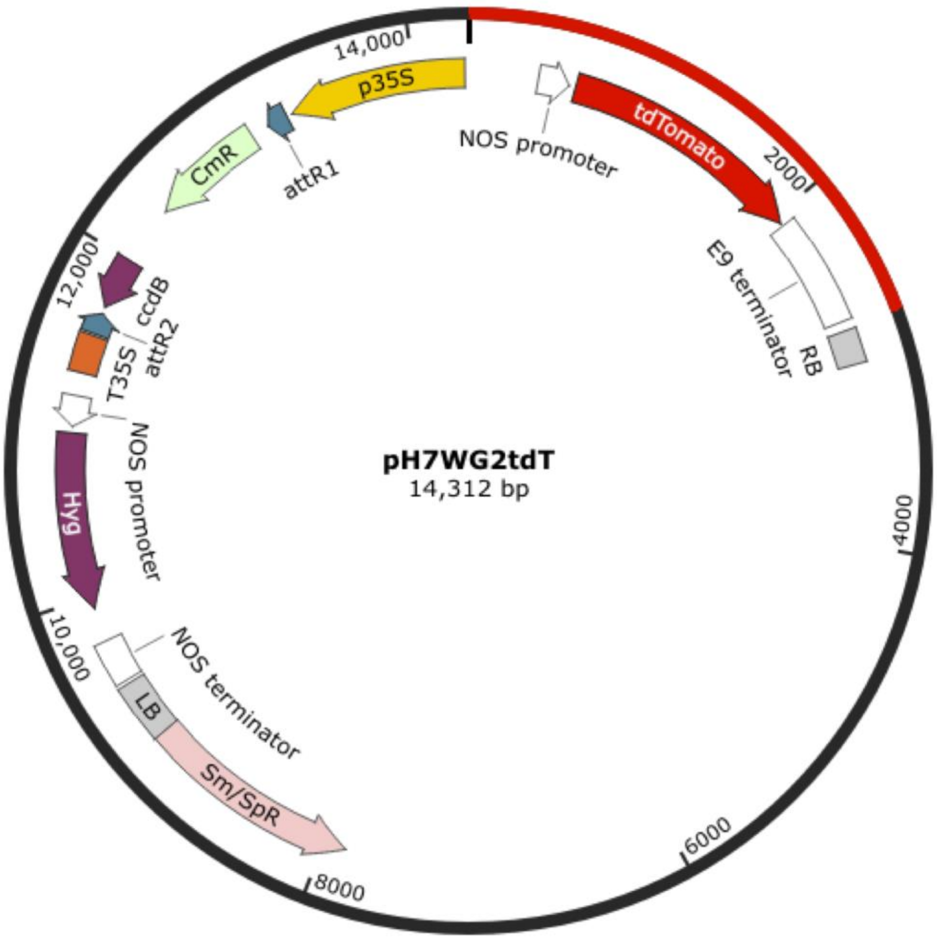

Figure S11. *In silico* map of the pH7WG2tdT vector. This was derived from the pH7WG2D vector (Karimi *et al.*, 2002); the p35S::EgfpER::35ST cassette was replaced by pNOS::tdTomato::E9T obtained from the pTDT-DC-RNAi vector [75]. Image was created with SnapGene version 2.3.2. software.

Supplementary Tables

Table S1. Size of the *ADF* gene family in various plants

**Plant type**	**Organism**	**Number of ADF members**
Monocot	*Oryza sativa*	9
	Zea mays	14
Non-legume dicots	*Arabidopsis thaliana*	11
Legumes	*Glycine max*	18
	Lotus japonicus	10
	Medicago truncatula	8
	Phaseolus vulgaris	9
	Vigna unguiculata	7

Table S2. Percentage of nucleotide sequence identity among *P. vulgaris ADF* genes. Pairwise sequence nucleotide alignment was performed using the EMBOSS Needle tool. (http://www.ebi.ac.uk/Tools/psa/emboss_needle/nucleotide.html)

	PvADFA	*PvADFB*	*PvADFC*	*PvADFD*	*PvADFE*	*PvADFF*	*PvADFG*	*PvADFH*	*PvADFI*
PvADFA	100								
PvADFB	77.9	100							
PvADFC	57.8	62.9	100						
PvADFD	73.6	74.0	55.0	100					
PvADFE	70.2	70.5	52.1	84.0	100				
PvADFF	62.4	62.8	51.2	63.7	61.0	100			
PvADFG	62.4	60.6	48.9	61.4	60.2	63.3	100		
PvADFH	60.6	57.8	49.6	65.1	62.9	61.1	77.3	100	
PvADFI	60.0	63.6	49.6	62.6	59.6	85.2	64.2	67.3	100

Table S3. Percentage of amino acid sequence identity between PvADF and AthADF proteins. Pairwise protein alignment was performed using the EMBOSS Needle tool (http://www.ebi.ac.uk/Tools/psa/emboss_needle/).

	PvADFA	PvADFB	PvADFC	PvADFD	PvADFE	PvADFF	PvADFG	PvADFH	PvADFI
PvADFA	100								
PvADFB	80.4	100							
PvADFC	60.3	60.7	100						
PvADFD	76.4	75.5	57.3	100					
PvADFE	70.7	68.3	54.1	84.2	100				
PvADFF	55.6	53.8	40.0	57.3	52.4	100			
PvADFG	55.4	57.8	44.9	61.9	55.1	57.0	100		
PvADFH	56.5	58.2	42.7	58.9	54.1	60.1	79.6	100	
PvADFI	55.6	55.2	41.6	57.3	53.1	89.5	61.1	65.5	100
AthADF1	74.3	70.5	54.6	86.3	**82.0**	52.4	59.2	57.5	55.2
AthADF2	74.6	72.3	55.7	85.6	79.1	53.8	57.1	58.2	56.6
AthADF3	68.6	69.1	51.9	80.6	82	51.0	53.7	56.2	53.8
AthADF4	75.0	71.2	55.1	88.5	83.5	52.4	59.9	57.5	55.9
AthADF5	54.2	53.1	39.5	53.1	49.0	83.9	56.4	58.8	86.7
AthADF6	56.5	55.5	41.7	57.5	52.7	57.4	78.9	78.8	58.8
AthADF7	84.8	80.3	59.0	77.0	69.8	55.9	56.5	58.2	56.6
AthADF8	75.7	74.3	53.8	75.7	70.7	54.1	56.8	53.7	55.5
AthADF9	51.4	51.8	36.9	54.6	51.8	73.4	55.1	61.6	79.7
AthADF10	81.9	79.6	57.9	76.3	70.5	52.4	55.8	56.2	53.1
AthADF11	78.6	75.7	55.4	77.1	70.0	54.8	56.8	54.4	56.2

Table S4. Annotation of ADFs aminoacid sequences used for the phylogenetic analysis.

Organism	Protein name	Annotation	Database
H.sapiens	HumanCofilin/ADF1	NP_005498.1	NCBI
A. thaliana	AthADF1	AT3G46010.2	Phytozome
	AthADF2	AT3G46000.1	Phytozome
	AthADF3	AT5G59880.1	Phytozome
	AthADF4	AT5G59890.1	Phytozome
	AthADF5	AT2G16700.1	Phytozome
	AthADF6	AT2G31200.1	Phytozome
	AthADF7	AT4G25590.1	Phytozome
	AthADF8	AT4G00680.1	Phytozome
	AthADF9	AT4G34970.1	Phytozome
	AthADF10	AT5G52360.1	Phytozome
	AthADF11	AT1G01750.1	Phytozome
P. vulgaris	PvADFA	Phvul.007G070500.1	Phytozome
	PvADFB	Phvul.002G156700.1	Phytozome
	PvADFC	Phvul.002G288100.1	Phytozome
	PvADFD	Phvul.007G157800.1	Phytozome
	PvADFE	Phvul.006G132700.1	Phytozome
	PvADFF	Phvul.009G120100.1	Phytozome
	PvADFG	Phvul.007G108800.1	Phytozome
	PvADFH	Phvul.001G160700.1	Phytozome
	PvADFI	Phvul.011G034600.1	Phytozome
G.max	GmADF53	Glyma.15G125300.1	Phytozome
	GmADF92	Glyma.09G019200.1	Phytozome
	GmADF17	Glyma.13G131700.1	Phytozome
	GmADF40	Glyma.10G044000.2	Phytozome
	GmADF65	Glyma.05G206500.1	Phytozome
	GmADF45	Glyma.11G024500.1	Phytozome
	GmADF89	Glyma.01G218900.1	Phytozome
	GmADF55	Glyma.10G235500.1	Phytozome
	GmADF58	Glyma.20G158900.1	Phytozome
	GmADF34	Glyma.08G013400.1	Phytozome
	GmADF44	Glyma.19G164400.1	Phytozome
	GmADF29	Glyma.03G162900.1	Phytozome
	GmADF98	Glyma.20G209800.1	Phytozome
	GmADF31	Glyma.12G031700.1	Phytozome
	GmADF39	Glyma.06G003900.1	Phytozome
	GmADF66	Glyma.11G106600.1	Phytozome
	GmADF33	Glyma.06G033400.1	Phytozome
	GmADF80	Glyma.10G180700.1	Phytozome
M. truncatula	MtADF67	Medtr2g028670.1	Phytozome
	MtADF21	Medtr8g088210.1	Phytozome
	MtADF95	Medtr1g068950.1	Phytozome
	MtADF47	Medtr8g098470.1	Phytozome
	MtADF43	Medtr5g010430.1	Phytozome
	MtADF89	Medtr7g096890.1	Phytozome
	MtADF59	Medtr4g073590.1	Phytozome
	MtADF17	Medtr1g076170.1	Phytozome

Z. mays	ZmADF40	GRMZM2G037140_T01	Phytozome
	ZmADF22	GRMZM2G097122_T01	Phytozome
	ZmADF71	GRMZM2G463471_T01	Phytozome
	ZmADF27	GRMZM2G071327_T01	Phytozome
	ZmADF12	GRMZM2G015127_T01	Phytozome
	ZmADF03	GRMZM2G117603_T01	Phytozome
	ZmADF78	GRMZM2G130678_T01	Phytozome
	ZmADF25	GRMZM2G002825_T01	Phytozome
	ZmADF02	GRMZM2G060702_T03	Phytozome
	ZmADF42	GRMZM2G077942_T01	Phytozome
	ZmADF07	GRMZM2G108807_T01	Phytozome
	ZmADF75	GRMZM2G147775_T01	Phytozome
	ZmADF87	GRMZM2G064875_T01	Phytozome
	ZmADF33	GRMZM2G108833_T01	Phytozome
O. sativa	OsADF47	LOC_Os02g44470.1	Phytozome
	OsADF91	LOC_Os04g46910.1	Phytozome
	OsADF34	LOC_Os12g43340.1	Phytozome
	OsADF79	LOC_Os03g56790.1	Phytozome
	OsADF58	LOC_Os03g60580.1	Phytozome
	OsADF95	LOC_Os03g13950.1	Phytozome
	OsADF67	LOC_Os10g37670.1	Phytozome
	OsADF09	LOC_Os07g30090.2	Phytozome
	OsADF17	LOC_Os07g20170.1	Phytozome
L.japonicus	LjADF90	LjTC58090.[121:531].sp.tr	LIS (legumeinfo.org)
	LjADF30	Lj-TC59530.[90:506].sp.tr	LIS (legumeinfo.org)
	LjADF59	Lj-FS345159.[182:592].sp.tr	LIS (legumeinfo.org)
	LjADF18	Lj-TC58418.[117:609].sp.tr	LIS (legumeinfo.org)
	LjADF98	LjNEST98d3r.[131:511].sp.tr	LIS (legumeinfo.org)
	LjADF83	LjTC60283.[126:542].sp.tr	LIS (legumeinfo.org)
	LjADF50	LjTC60150.[110:520].sp.tr	LIS (legumeinfo.org)
	LjADF47	LjTC60947.[91:501].sp.tr	LIS (legumeinfo.org)
	LjADF85	LjTC62885.[144:560].sp.tr	LIS (legumeinfo.org)
	LjADF84	LjTC63884.[105:521].sp.tr	LIS (legumeinfo.org)
V. unguiculata	VuADF84	VuTC14684.[145:561].sp.tr	CGKB
	VuADF38	VuTC1238.[179:595].sp.tr	CGKB
	VuADF07	VuUCRVU07_CCNP7094_b1.[41:334INCOM	CGKB
	VuADF98	VuTC2698.[85:501].sp.tr	CGKB
	VuADF93	VuTC6693.86:502.sp.tr	CGKB
	VuADF08	VuUCRVU08_CCNS3648_b1.[20:436].sp.tr	CGKB
	VuADF02	VuTC2602.[96:512].sp.tr	CGKB

Table S5. Gene-specific oligonucleotides used

**Name**	**Sequence 5′ – 3′**	**Amplicon size (bp)**
Ef1α-Up	GGTCATTGGTCATGTCGACTCTGG	146
Ef1α-Lw	GCACCCAGGCATACTTGAATGACC	
*Pv*ADFE-OE-Up	GCTCCACCACACCACAGTT	833
*Pv*ADFE-OE-Lw	TTCAACTAGTATTGGATAAAAGACCAC	
*Pv*ADFE-RNAi-Up	GTACGCTTTCTGGTGGGAGCAC	355
*Pv*ADFE-RNAi-Lw	ACAAAAGAAAGCATATATCGTCCAAA	
p*Pv*ADFE-Up	TGCACCTATGCTTGTCTCCTACAC	1383
p*Pv*ADFE-Lw	GGTGATGACGATGGTGTTGGG	
*Pv*ADFA-qPCR-Up	ACAGCTAGCTTTGGCGGCAC	91
*Pv*ADFA-qPCR-Lw	GGTTTACAATGTAGGCCAGTTGAC	
*Pv*ADFB-qPCR-Up	GTCCTCCCTTTTGTTGTCTCAAC	142
*Pv*ADFB-qPCR-Lw	GTACATGCCATTTTGGATTTGTCG	
*Pv*ADFC-qPCR-Up	TGGGAGCCATCTTTCTTTGCC	108
*Pv*ADFC-qPCR-Lw	CTGATAAGGATCGGTACAAGGAAG	
*Pv*ADFD-qPCR-Up	TCTTCCACCTCAAAACCCTTT	133
*Pv*ADFD-qPCR-Lw	AGTCATCGTGGACAGCCATAC	
*Pv*ADFE-qPCR-Up	GCTCCACCACACCACAGTTTTC	154
*Pv*ADFE-qPCR-Lw	GGTGATGACGATGGTGTTGGG	
*Pv*ADFF-qPCR-Up	TATAGGGCCAGCTGTTGCTCTCA	91
*Pv*ADFF-qPCR-Lw	CATCTTGAAAGCCATCGCCATT	
*Pv*ADFG-qPCR-Up	GGAGCTACCCAAGAGGGTCGTG	105
*Pv*ADFG-qPCR-Lw	CAGAGAAAGACCATAGTAGAACTAAAGG	
*Pv*ADFH-qPCR-Up	CATGCTTTATCATCTGCAGAGCCC	135
*Pv*ADFH-qPCR-Lw	CGCTCTATAACACAGGTTTAGCAAATTGG	
*Pv*ADFI-qPCR-Up	AAGTCGGGGAGATGGTGCTTAT	130
*Pv*ADFI-qPCR-Lw	TGTTATGTGGTGAGAAGCAGAACAAAG	
*Pv*NIN-qPCR-Up	GGGGATTCAGAGATTTGCAG	101
*Pv*NIN-qPCR-Lw	AACCCACTCTTGAGCATCGT	
*Pv*ENOD2-qPCR-Up	AGTGTACACACCCCCACCATACCA	137
*Pv*ENOD2-qPCR-Lw	TCTTGGATGGTGGATAGTGGCCA	
*Pv*CyclinB-qPCR-Up	GGATTGCGCCAAAAACCTAGT	135
*Pv*CyclinB-qPCR-Lw	AGTGTTGTCAAGTGCTTTGCTGGAG	
